# CDC7 kinase (DDK) inhibition disrupts DNA replication leading to mitotic catastrophe in Ewing sarcoma

**DOI:** 10.1038/s41420-022-00877-x

**Published:** 2022-02-26

**Authors:** Jeffrey C. Martin, Jennie R. Sims, Ajay Gupta, Tamara J. Hagoel, Lingqiu Gao, Miranda L. Lynch, Anna Woloszynska, Thomas Melendy, Jeremy F. Kane, Joseph Kuechle, Joyce E. Ohm

**Affiliations:** 1 Department of Cancer Genetics and Genomics, Roswell Park Comprehensive Cancer Center, Buffalo, NY USA; 2 Division of Pediatric Oncology, Roswell Park Comprehensive Cancer Center, Department of Pediatrics, University at Buffalo Jacobs School of Medicine and Biomedical Sciences, Buffalo, NY USA; 3 Hauptman-Woodward Medical Research Institute, Buffalo, NY USA; 4 Department of Pharmacology & Therapeutics, Roswell Park Comprehensive Cancer Center, Buffalo, NY USA; 5 Department of Microbiology and Immunology, State University of New York at Buffalo, Buffalo, NY USA; 6 Department of Surgery, Roswell Park Comprehensive Cancer Center, Buffalo, NY USA

**Keywords:** Cancer genomics, DNA damage checkpoints, Stalled forks

## Abstract

Ewing sarcoma is the second most common bone malignancy in children and adolescents. In recent years, a large body of evidence has emerged that suggests Ewing tumors harbor large amounts of replication stress (RS). CDC7, also known as DDK (DBF4-dependent kinase), is a serine/threonine kinase that is involved in a diverse array of cellular functions including the regulation of DNA replication initiation and activation of the RS response. Due to DDK’s diverse roles during replication, coupled with the fact that there is an increased level of RS within Ewing tumors, we hypothesized that Ewing sarcoma cells would be particularly vulnerable to DDK inhibition. Here, we report that DDK inhibition resulted a significant reduction in cell viability and the induction of apoptosis, specifically in Ewing sarcoma cells. Treatment with DDK inhibitors dramatically reduced the rate of replication, prolonged S-phase, and led to a pronounced increase in phospho-CDC2 (Y15), indicating delay of mitotic entry. The induction of cell death corresponded to mitotic exit and G1 entry, suggesting improper mitotic progression. In accordance with this, we find that DDK inhibition caused premature mitotic entry resulting in mitotic abnormalities such as anaphase bridges, lagging chromosomes, and cells with >2 poles in Ewing sarcoma cells. This abnormal progression through mitosis resulted in mitotic catastrophe as evidenced by the formation of micronuclei and induction of DNA damage. Together, these findings suggest that DDK activity is required for the faithful and timely completion of DNA replication in Ewing cells and that DDK inhibition may present a viable therapeutic strategy for the treatment of Ewing sarcoma.

## Introduction

Ewing sarcoma is a rare childhood sarcoma that mainly presents in the bone [1, 2]. It is characterized by a recurrent chromosomal translocation between the RNA-binding protein, EWSR1, and the ETS transcription factor, FLI1 [3]. Treatment of Ewing sarcoma has remained largely unchanged for several decades, consisting of surgical resection and the use of alkylating agents, such as Ifosfamide and Cyclophosphamide, and topoisomerase poisons, mainly Etoposide and Doxorubicin [4]. This chemotherapy regimen is associated with severe side effects [2] and there is a desperate need for the development of novel therapeutic strategies to effectively treat Ewing sarcoma while limiting toxicity.

Ewing tumors have been previously shown to harbor elevated levels of endogenous DNA damage, elevated levels of replication stress (RS) and sensitivity to ATR [5, 6] and CHK1 inhibitors [7, 8]. This endogenous replication stress has recently been attributed to the formation of R-loops due to an EWS-FLI1-dependent promotion of CDK9-mediated RNA Pol-II activation [9]. The formation of R-loops disrupts the progression of the replisome and results in fork stalling and the activation of ATR [10]. Also, low dose treatment with replication inhibiting agents such as hydroxyurea (depletes dNTPs) and aphidicolin (inhibits DNA polymerase) induces DNA damage and cell death in Ewing cells [8]. Together these observations indicate that Ewing sarcoma cells are dependent on their ability to successfully manage high levels of replication stress.

An important RS response kinase, DDK (DBF4-dependent kinase), also known as CDC7 (cell division cycle 7) is a serine/threonine kinase that has been shown to play diverse roles during S-phase [11–16]. Its main role is in the activation of replication origins and initiation of DNA replication. In conjunction with CDK2-cyclinA, DDK directly phosphorylates the MCM helicase complex, promoting replication initiation [17]. Therefore, DDK is considered a vital kinase during S-phase as it functions at the center of the DNA replication program. In the context of RS, DDK’s role at replication origins is required for the use of dormant origins, a vital component of the RS response [18, 19]. DDK is also required for the full activation of ATR and CHK1 and for activation of the S-phase checkpoint through various mechanisms [13, 15]. In this sense, DDK function promotes faithful DNA replication. On the other hand, unchecked DDK activity can allow for rapid completion of DNA replication and an increased capacity for cellular division [20]. This increases the potential for rapid consumption of nucleotides and exhaustion of replication co-factors resulting in wide-scale replication fork stalling [11]. Also, an elevated number of active replication forks would, by definition, leave cells vulnerable to intrinsic and extrinsic sources of fork stalling. For these reasons, DDK activity needs to be closely monitored throughout S-phase. In rapidly dividing cells, especially those with high levels of intrinsic RS, replication fidelity and cell division rely on the ability to readily activate/deactivate DDK depending on the nuclear environment and requirements for replication [11]. Due to the elevated level of RS within Ewing tumors and the central role of DDK in the RS response, we hypothesized that Ewing cells would be highly sensitive to DDK inhibition.

## Materials and methods

### Cell lines

The Ewing sarcoma cell lines that were used were A673, SK-ES-1, and RD-ES (ATCC). As a control non-Ewing sarcoma cancer cell line, the osteosarcoma cell line U2OS was used. A673 cells were cultured in Dulbecco’s modified Eagle’s medium (DMEM; Corning. Mediatech Inc.) supplemented with 10% fetal bovine serum (FBS) and 1% antibiotic-antimycotic (Gibco Life Technologies). U2OS cells were cultured in McCoy’s 5 A (Corning. Mediatech Inc.) supplemented with 10% FBS and 1% antibiotic-antimycotic (Gibco Life Technologies). SK-ES-1 cells were cultured in McCoy’s 5 A medium (Corning. Mediatech Inc.) supplemented with 15% FBS and 1% antibiotic-antimycotic (Gibco Life Technologies). RD-ES cells were cultured in RPMI-1640 (Corning. Mediatech Inc.) supplemented with 15% FBS and 1% antibiotic-antimycotic (Gibco Life Technologies). BJ cells were cultured in Minimum Essential Media (MEM; Corning. Mediatech Inc.) supplemented with 10% FBS and 1% antibiotic-antimycotic (Gibco Life Technologies). All cell lines were grown at 37 °C and 5% CO_2_.

### Chemical Inibitors

The CDC7 kinase inhibitor, XL413, was purchased from Sigma-Aldrich (cat. No. SML1401; batch #: 0000036390) and dissolved in sterile-filtered ddH_2_O to a final concentration of 5 mM. The CDC7 kinase inhibitor TAK-931 (Simurosertib) was purchased from MedChemExpress (MCE) (cat. No. HY-100888) as a 10 mM stock dissolved in DMSO.

### Cell viability

Cells were seeded at appropriate densities (adherent cell lines: 5,000 cells per well; mixed-adherent cell lines: 10,000 cells per well) and allowed to incubate overnight at 37 °C. Cells were treated with serial dilutions of the drug and incubated for 72 h. Cell counting kit-8 (WST-8/CCK8) reagent from GLPBIO was added to each well and allowed to incubate for 2 h. The absorbance was measured at 450 nm on a SpectraMax M2/M2E microplate reader (Molecular Devices). Final cell viability was determined relative to the average absorbance of solvent control-treated cells.

### Western blotting

Cell lysates were separated by SDS-PAGE and then transferred to polyvinylidenedifluoride (PVDF) membranes. The membranes were blocked with 5% milk in TBS-T (TBS containing 0.1% Tween-20) for 1 h at room temperature and then incubated in 1° antibody dilutions overnight at 4 °C: rabbit anti-Cleaved PARP (Asp-214) #9451 (9541 S), 1:1000; rabbit anti-MCM2 #4007 (4007 S), 1:1000; Cell Signaling Technology, Inc; rabbit anti-GAPDH, ab9485; 1:5000; rabbit anti-gamma H2A.X (phospho S139), ab11174, 1:1000; rabbit anti-MCM2 (phospho S40 + S41), ab70371, 1:1000; Abcam plc; rabbit anti-Phospho-Histone H3 (Ser10), Cell signaling technology Cat No.: 9701 S, 1:1000; rabbit anti-phospho-CDC2(Y15), Cell Signaling Technology Cat No.: 9111, 1:1000; rabbit anti-CDC2, Cell Signaling Technology, Cat No.: 77055, 1:1000; mouse anti-CDT1, Santa Cruz Biotechnology, Cat No.: sc-365305, 1:1000; rabbit anti-Cyclin B1, Cell Signaling Technology, Cat No.: 4138, 1:1000; rabbit anticleaved-Caspase 3 (Asp175), Cell Signaling Technology Cat No.: 9661, 1:1000; mouse anti-PARP1, Santa Cruz Biotechnology, Cat No.: sc-8007. All original western blot images are included as Supplementary Data.

The following day, the membranes were washed 3 times for 10 min each in TBS-T and then incubated with 2° antibodies for 1 h at room temperature: Goat anti-Rabbit IgG (H + L) Highly cross absorbed Secondary Antibody, Alexa Fluor Plus 800, Invitrogen #A32735, 1:40,000; Goat anti-Rabbit IgG (H + L) Highly cross absorbed Secondary Antibody, Alexa Fluor Plus 680, Invitrogen #A32734, 1:40,000; Goat anti-Mouse IgG (H + L) Highly cross absorbed Secondary Antibody, Alexa Fluor Plus 800, Invitrogen #A32730, 1:40,000; Goat anti-Mouse (H + L) Highly cross absorbed Secondary Antibody, Alexa Fluor Plus 680, Invitrogen #A32729, 1:40,000. Proteins were detected using an Odyssey western blot imaging system by LI-COR Biosciences. GAPDH was used as an internal loading control.

### Immunofluorescence microscopy

Cells were seeded onto EtOH-sterilized glass coverslips and allowed to attach for 24-48 h. Following drug treatment, media was removed, and coverslips were washed in 1X PBS. For EdU incorporation assays, 10 µM EdU was added to media 30 min prior to cell fixation. Cells were fixed with 4% paraformaldehyde for 15 min and permeabilized with 0.05% Triton-X100 for 15 min at room temperature. For EdU incorporation assays, the click-It reaction was performed according to manufacturer’s instructions (Click-IT^TM^ EdU Cell Proliferation Kit for Imaging, Alexa Fluor^TM^ 488 dye, Cat. No. C10337). Coverslips when then blocked with 5% milk for 1 h at room temperature and then incubated with 1° antibodies overnight at 4 °C protected from light: rabbit anti-gamma H2A.X (phospho S139), ab11174, 1:500; rabbit anti-53BP1, ab36823, 1:500. Following the overnight incubation, coverslips were washed with 1X PBS for 10 min, 3 times and then incubated with 2° antibodies for 1 h at room temperature protected from light: Goat Anti-Mouse IgG H&L (Alexa Fluor® 488) pre-absorbed, ab150117, 1:500; Goat Anti-Rabbit IgG H&L (Alexa Fluor® 488), ab150077, 1:500; Abcam plc. Goat anti-Mouse IgG (H + L) Cross-Absorbed Secondary Antibody, Alexa Fluor 568, A-11004, 1:500; Goat anti-Rabbit IgG (H + L) Cross-Absorbed Secondary Antibody, Alexa Fluor 568, A-11011, 1:500; ThermoFisher Scientific. The coverslips were then washed with PBS for 10 min, 3 times, stained with DAPI, and mounted using ProLong^TM^ Gold Antifade Mounting reagent (ThermoFisher Scientific, Cat no. P36930). Fluorescent images were captured on a Zeiss Axio Imager.A2 at 63x magnification. The corrected gamma-H2AX intensity and 53BP1 foci per cell for individual cells were measured using ImageJ software.

### Cell cycle

Cells were treated and then fixed in ice-cold 70% ethanol at 4 °C for 30 min. Cells were washed with 1X PBS and then incubated with Propidium Iodine/RNase solution for 2 h. DNA content was then analyzed using flow cytometry. ModFit 5.0 software was used to analyze cell cycle based on DNA content. For 2-dimensional cell cycle analysis, cells were incubated with 10 µM EdU for 1 h prior to fixation and DNA stain. EdU was stained using Click-It EdU AlexaFLuor488 Flow Cytometry Assay Kit from Thermo Fisher Scientific (Cat No.: C10425) and followed as per manufacturer’s protocol.

### Annexin-V staining

Following drug exposure, cells were trypsinized, washed with PBS, and resuspended in Annexin-binding buffer. Apoptotic cells were stained for annexin-V and DNA of dead cells was stained with propidium iodide according to the manufacturer’s instructions (Dead Cell Apoptosis Kit with Annexin V FITC and PI, for flow cytometry, ThermoFisher Scientific, Cat. No.: V13242). Cells were then analyzed using flow cytometry.

### Phospho-Histone H3 staining

Following treatment, cells were fixed in appropriate volume of 4% paraformaldehyde at room temperature for 15 min. Cells were then permeabilized by adding 100% ice-cold Methanol and incubating for 10 min on ice. Cells were then wash in PBS and resuspended in 100 µL of 1^o^ antibody (Phospho-Histone H3 (Ser10), Cell signaling technology Cat No.: 9701 S, 1:50 dilution) for 1 h at room temperature. Cells were then washed with PBS and resuspended in 100 µL of diluted secondary antibody (Goat Anti-Mouse IgG H&L (Alexa Fluor® 488) pre-absorbed, ab150117, 1:500; Goat Anti-Rabbit IgG H&L (Alexa Fluor® 488), ab150077, 1:500; Abcam plc. Goat anti-Mouse IgG (H + L) Cross-Absorbed Secondary Antibody, Alexa Fluor 568, A-11004, 1:500; Goat anti-Rabbit IgG (H + L) Cross-Absorbed Secondary Antibody, Alexa Fluor 568, A-11011, 1:500; ThermoFisher Scientific). DNA content was labeled with propidium iodide as described in methods description of “cell cycle”. Cells were then analyzed using flow cytometry.

### Double-thymidine block

Cells were treated with 2 mM Thymidine (Millipore Sigma, Cat. Num.: T9250) for 18 h. Thymidine was then removed, and cells were washed several times with PBS. Media was replaced with Thymidine-free full serum media for 9 h. Cells were then treated with 2 mM Thymidine for an additional 18 h at which point, Thymidine was washed out and media was replaced + /- specified inhibitors for varying amounts of time.

## Results

### DDK inhibition causes cell death in Ewing sarcoma cells

Due to the vital role that DDK plays during DNA replication, we hypothesized that Ewing sarcoma cells would be highly sensitive to its inhibition. To test this, we treated three Ewing sarcoma cell lines (A673, RD-ES, and SK-ES-1) with increasing concentrations of two independent DDK inhibitors, XL413 and TAK-931, for 72 h and assessed cell viability. TAK-931 has been shown to be a more specific inhibitor of DDK [21] and was recently tested in a phase 2 clinical trial for the treatment of metastatic pancreatic cancer, metastatic colorectal cancer, and advanced solid tumors [21]. The osteosarcoma cell line, U2OS, was used as a non-Ewing sarcoma/bone tumor cell line control to validate the unique responses within Ewing cells.

**Fig. 1:**
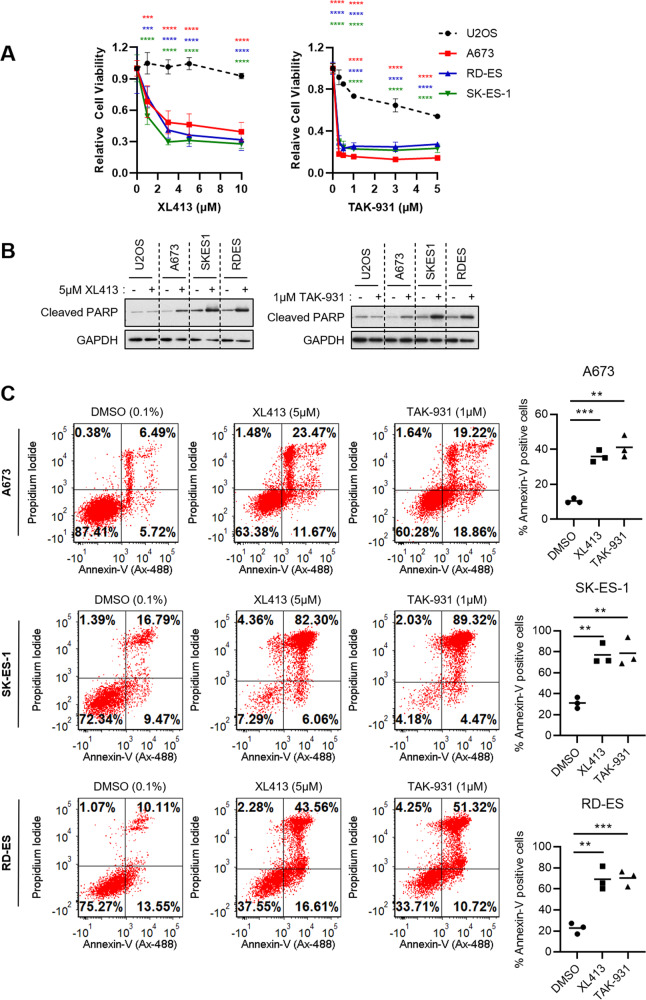
Ewing Sarcoma cells are sensitive to DDK inhibition. **A** Ewing Sarcoma cells (A673, RD-ES and SK-ES-1) and the non-Ewing, osteosarcoma cell line U2OS were treated with increasing concentrations of the DDK inhibitors XL413 and TAK-931 for 72 h and cell viability was measured using CCK-8 cell viability reagent. Relative viability was calculated based on DMSO treated cells (*n* = 3 biological replicates). 2-way ANOVA multiple comparisons, ***p < 0.001, ****p < 0.0001. **B** Cells were treated with either 0.1% DMSO, 5 µM XL413 (left panel) or 1 µM TAK-931 (right panel) for 48 h. Whole-cell lysates were collected, and western blot was performed for the specified proteins. All original western blot images are included in Supplementary Data. GAPDH was used as a loading control. **C** Ewing Sarcoma cells were treated with 0.1% DMSO, 5 µM XL413 or 1 µM TAK-931 for 48 h. Cells were stained with anti-Annexin-V (AlexaFluor-488 conjugated) and propidium iodide and analyzed using flow cytometry. Apoptotic cells were designated as Annexin-V (upper right and lower right quadrants) positive cells and were measured using FCS express v7 (*n* = 3 biological replicates). Bars represent the mean of the data. Unpaired *t* test, ***p < 0.001, **p < 0.01.

We found that Ewing sarcoma lines were significantly more sensitive to both DDK inhibitors as compared to the U2OS osteosarcoma cell line (Fig. 1A). To ensure this was a phenomenon shared among several Ewing sarcoma cell lines, a wider range of Ewing sarcoma cell lines with varying genetic backgrounds (TP53, CDKN2A, and STAG2 WT vs. mutant) were tested. Cells were treated with TAK-931 for 72 h and IC50 values were calculated (Fig. S1A). In our hands, all Ewing sarcoma cell lines tested had IC50s below that of U2OS (5.33 µM) (Fig. S1B, C). Importantly, there was no apparent relationship between TP53, STAG2, or CDKN2A status and response to TAK-931, suggesting that the basis for this sensitivity is independent of the function of these proteins (Fig. S1C-E).

To validate that the reduction in viability due to DDK inhibition was a result of programmed cell death and not simply a cytostatic phenomenon, apoptotic induction was measured. A clear accumulation of cleaved-PARP was observed in all three Ewing cell lines after treatment with both XL413 (Fig. 1B – left) and TAK-931 (Fig. 1B – right) indicative of caspase-mediated apoptotic induction whereas no accumulation of cleaved-PARP was observed in the U2OS cell line. These results were validated by annexin-V/PI staining that showed a statistically significant increase in apoptotic/dead cells upon treatment with both DDK inhibitors (Fig. 1C). These results support a model whereby DDK activity is required for the maintenance of Ewing sarcoma cell viability and its inhibition results in apoptosis in these cells.

### DDK inhibition disrupts DNA replication and cell cycle progression in Ewing sarcoma cells

**Fig. 2:**
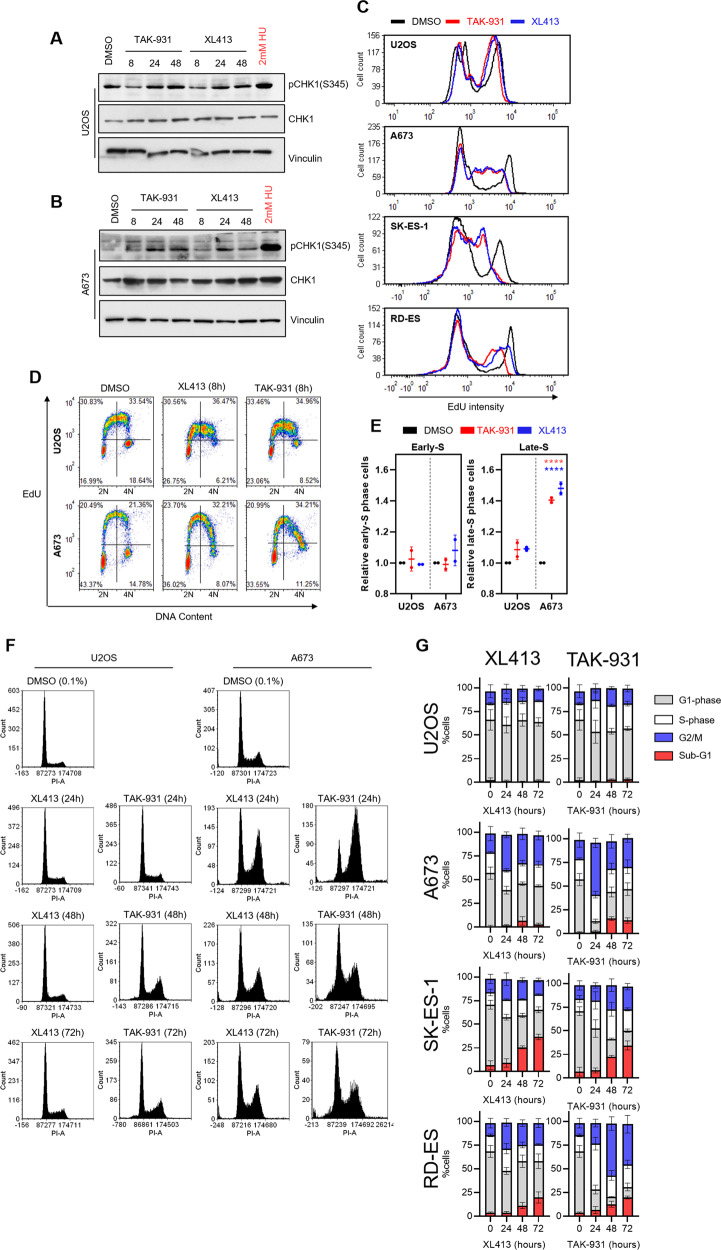
DDK inhibition disrupts DNA replication and alters cell cycle progression in Ewing sarcoma cells. **A, B** U2OS (**A**) and A673 (**B**) cells were treated with 1 µM XL413 or 300 nM TAK-931 for the indicated timepoints. Protein lysates were collected, and a western blot was performed to analyze the relative levels of the indicated proteins. 2 mM hydroxyurea (HU) was used as a positive control for replication stress induction. **C** Cells were treated with 0.1% DMSO, 1 µM XL413 or 300 nM TAK-931 for 8 h followed by a 1-h incubation with 10 µM EdU. Cells were then fixed and stained for EdU and relative EdU intensity was measured using FACS. Representative experiment of 3 biological replicates is shown. **D** U2OS and A673 cells were treated with either 0.1% DMSO, 1 µM XL413, or 300 nM TAK-931 for 8 h followed by a 1-h incubation with 10 µM EdU. Cells were then fixed and stained for EdU (Alexa-fluor 488) and DNA content (PI) and analyzed using FACS. Representative dot plots of 2 biological replicates are shown. **E** Quantification of panel **D**. Specifically, calculations were done by dividing the percentage of cells in the upper right-hand quadrant (late-S phase) by the total number of cells in the upper left and upper right quadrants (total S-phase cells), relative to DMSO treated control cells (*n* = 2 biological replicates) 2-way ANOVA Dunnett’s multiple comparison test, ****p < 0.0001. Bars represent mean and standard deviation. **F** Cells were treated with 1 µM XL413 or 300 nM TAK-931 for the indicated timepoints. DNA content was stained with PI and analyzed using FACS. Representative histograms of 3 biological replicates are shown. **G** Quantification of panel **F** (*n* = 3 biological replicates).

DDK has been shown to contribute to the alleviation of RS through several mechanisms [13–15]. It is therefore likely that an increase in RS upon DDK inhibition may be contributing to the cell death observed within Ewing sarcoma cells. ATR is the main kinase within the RS response and is a commonly activated upon even slight impediments to replication fork progression [22]. We found that, upon DDK inhibition, there was a slight but consistent increase in the pCHK1-S345 (ATR-dependent phosphorylation) in the Ewing sarcoma cells (Fig. 2A) especially at later time points (>8 h) but not in U2OS (Fig. 2B). This small level of CHK1 phosphorylation was surprising, considering that DDK has been shown to be required for full ATR activation [15]. In fact, 8-h DDK inhibition seemingly reduced this phosphorylation mark in the U2OS cells, consistent with DDK’s contribution to ATR-CHK1 activation.

ATR activation is typically a response to replication fork stalling [22]. Extensive replication fork stalling would result in a noticeable reduction to the overall rate of replication and can be measured by monitoring the incorporation of nucleotides. To determine the effect of DDK inhibition on replication rates, we measured the level of incorporation of the nucleotide analog EdU after 8-h DDK inhibition. As expected, in U2OS cells, both TAK-931 and XL413 treatment led to a small and uniform reduction in EdU incorporation as measured by flow cytometry (Fig. 2C - top). This is consistent with DDK’s role in replication origin activation [17]. However, the reduction in EdU incorporation was much more dramatic and non-uniform in all three Ewing sarcoma lines (Fig. 2C – lower three panels). Due to DDK’s known role within S-phase, we hypothesized that its inhibition is unlikely to induce acute replication fork stalling. Rather, there is likely to be an accumulation of stalled replication forks as replication progresses, as DDK activity is involved in stalled fork recovery and the activation of dormant origins [14, 15, 23]. This could be measured by examining disparities in DNA replication between late and early S phase. We observed an accumulation of low EdU incorporating cells in late S-phase upon DDK inhibition in the Ewing sarcoma cells and not within the U2OS cells (Fig. 2D, E). This result, in combination with our observation that pCHK1 accumulates upon DDK inhibition (Fig. 2A) suggests that DDK inhibition results in the progressive accumulation of RS over the course of several hours.

Cell cycle analysis revealed that the U2OS cells experienced minimal changes upon DDK inhibition at all timepoints tested. However, at 24 h of DDK inhibition, the Ewing sarcoma cells appeared to accumulate in late-S and G2 (Fig. 2F, G), which is consistent with the reduction in EdU incorporation seen in Fig. 2C-E. Interestingly, there was a reduction in this population by 48 and 72 h accompanied by the emergence of sub-G1 DNA content, suggesting cell death (Fig. 2F, G – red bars). Similar results were seen in the SK-ES-1 and RD-ES cell lines (Fig. 2G and Fig. S2). Overall, these results suggest that DDK plays a large role in the conservation of DNA replication rates and S-phase progression in Ewing cells.

### DDK inhibition delays S-phase progression and mitotic entry in Ewing sarcoma cells

**Fig. 3:**
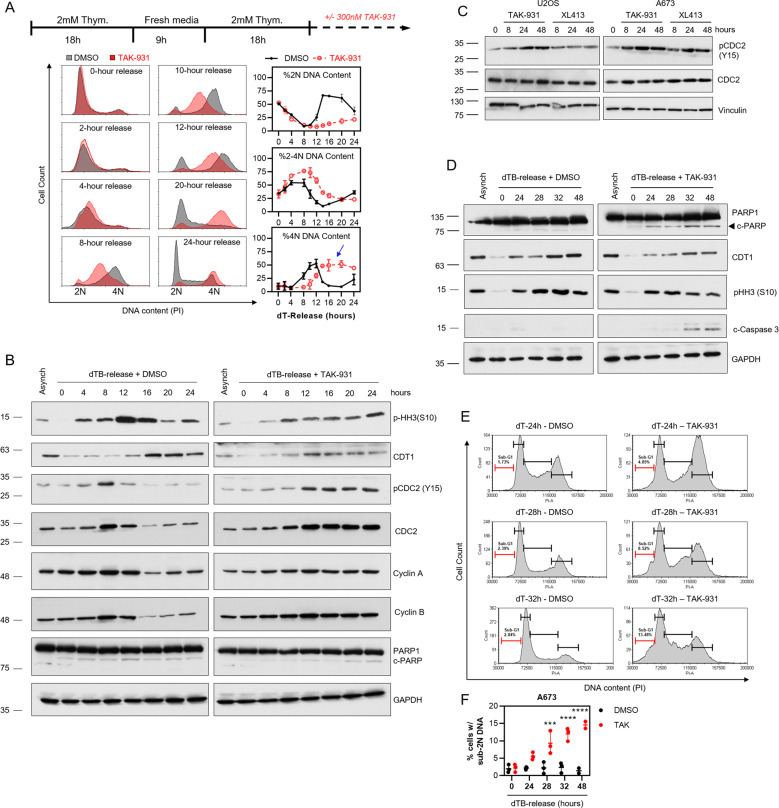
DDK inhibition prolongs S-phase and delays mitotic entry in Ewing sarcoma cells. **A** A673 cells were subjected to a double-thymidine block (dTB) and then released into S-phase in the presence of either 0.1% DMSO or 300 nM TAK-931 for the indicated timepoints. DNA content was stained with PI and then analyzed using FACS (*n* = 3 biological replicates). **B** A673 cells were subjected to a dTB and then released in the presence of 0.1% DMSO or 300 nM TAK-931 for the indicated timepoints. Protein was collected and a western blot was performed to analyze the protein levels of the indicated proteins. **C** U2OS and A673 cells were treated with 1 µM XL413 or 300 nM TAK-931 for the indicated timepoints. Protein was collected and a western blot was performed to analyze the protein levels of the indicated proteins. **D** A673 cells were subjected to a dTB and released into S-phase in the presence of either 0.1% DMSO or 300 nM TAK-931 for the indicated timepoints and protein was collected. **E** A673 cells were treated with 0.1% DMSO or 300 nM TAK-931 upon dTB release for the indicated timepoints. Cells were fixed and total DNA content was stained with PI. Representative histograms of 3 biological replicates are shown. **F** Quantification of sub-G1 DNA content from panel **D** (*n* = 3 biological replicates) 2-way ANOVA *** *p* = 0.0002, **** *p* < 0.0001.

To gain more insight into the effects of DDK inhibition on S-phase progression and mitotic entry/progression in Ewing sarcoma, A673 cells were synchronized at the beginning of S-phase using a double-thymidine block (dTB). The cells were then released into S-phase in the presence or absence of 300 nM TAK-931 and cell cycle progression was analyzed using immunoblot and FACS over the course of several days (Fig. 3A). In agreement with the data in Fig. 2, DDK inhibition resulted in a significant accumulation of cells with 2N-4N DNA content (Fig. 3A - %2N-4N DNA content) and elevated levels of cyclin A1 at late time points (>16 h upon dTB-release) (Fig. 3B). Cyclin A1 levels peak around 8-12 h post-dTB-release followed by a dramatic reduction by 16 h in the DMSO treated cells (Fig. 3B – left). In contrast to this, in the population of cells treated with TAK-931, Cyclin A1 levels never appear to diminish, even at late timepoints (>16 h) (Fig. 3B – right) suggesting an extension of late-S phase. Importantly, DMSO treated cells appeared to fully complete replication between 8-12 h upon dT-release while TAK-931 treated cells did not complete replication until ~20 h (Fig. 3A - %4 N DNA content) suggesting a delay in replication completion. This was confirmed by the observation that there was a synchronous entry into mitosis around 12 h in the DMSO treated population as was measured by phosphorylation of histone H3S10 (pHH3). (Fig. 3B - left). No such expression pattern was observed in the TAK-931 treated population. Instead, mitotic entry appeared to be less uniform, with the abundance of pHH3 being relatively consistent across most time points ranging from 8-20 h (Fig. 3B - right) with no clear peak at any timepoint. Importantly, cells seemed to reside in G2/M for a prolonged period compared to the DMSO treated cells (Fig. 3A – %4 N DNA content, blue arrow), suggesting delayed mitotic entry/progression after TAK-931 treatment. In agreement with this, the inhibitory phosphorylation of CDC2, the kinase responsible for mitotic progression [24], at tyrosine 15 was significantly more pronounced upon DDK inhibition as cells approached S-phase completion (~12 h), indicating a molecular inhibition of mitotic entry (Fig. 3B – right). This was accompanied by prolonged expression of the mitotic cyclin, cyclin B, further suggesting a delay in mitotic entry/progression upon DDK inhibition. Importantly, while TAK-931 treatment appeared to increase pCDC2-Y15 in the U2OS cells, this was significantly more pronounced in the A673 cells with both TAK-931 and XL413 treatment at all timepoints tested, suggesting a unique molecular response to DDK inhibition within Ewing sarcoma cells (Fig. 3C).

The delay in mitotic entry/progression in conjunction with the appearance of sub-G1 DNA content at late time points prompted us to hypothesize that DDKi-induced cell death in Ewing cells occurs during progression through mitosis. In agreement with this hypothesis, we did not observe evidence of apoptosis (c-PARP1, c-Caspase 3, and %sub-G1 DNA content) until late dTB-release timepoints upon DDK inhibition (24h–48h) (Fig. 3D-F). Instead, apoptotic markers appeared to peak around 32–48 h upon dTB-release, matching the expression pattern of the late-M/G1 protein CDT1 and inversely with the expression of pHH3 (Fig. 3D). In line with previous studies [21, 25] these data suggest that DDKi-induced apoptosis is occurring either during mitosis or as cells exit mitosis and enter G1.

### DDK inhibition causes abnormal mitosis in Ewing sarcoma cells

**Fig. 4:**
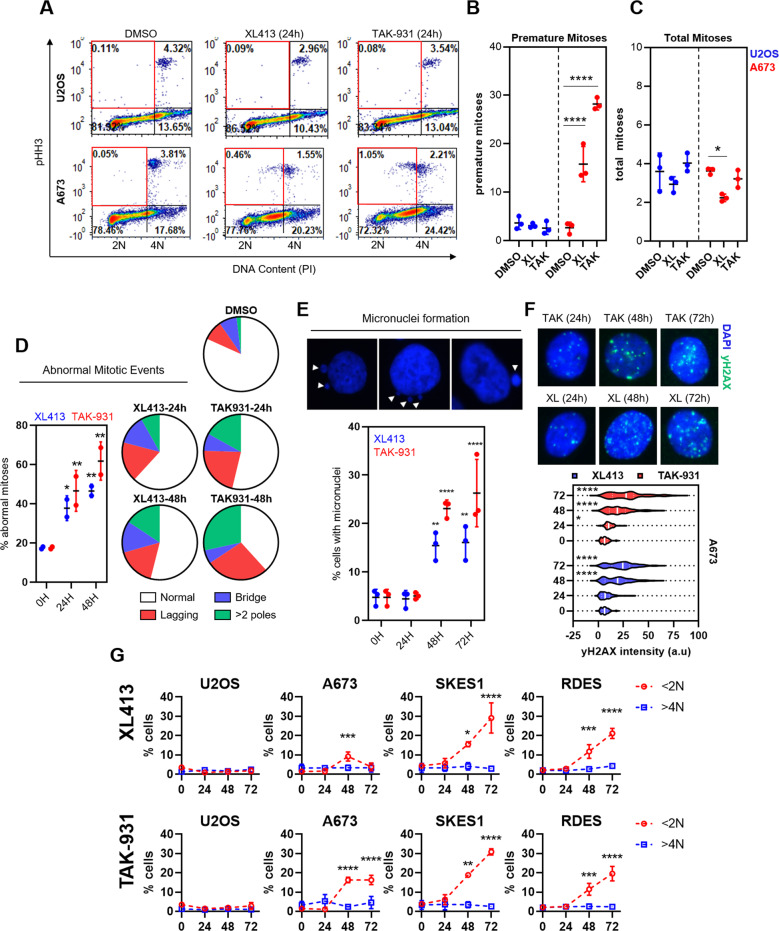
DDK inhibition causes premature mitotic entry and mitotic catastrophe in Ewing sarcoma cells. **A** A673 and U2OS cells were treated with 0.1% DMSO, 1 µM XL413 or 300 nM TAK-931 for 24 h. Cells were then stained for phospho-histone H3 (Serine 10) (pHH3) and DNA (propidium iodide) and analyzed using FACS (representative dot plots of 3 biological replicates). **B** Quantification of panel **A** of proportion of pHH3-positive cells that had <4 N DNA content per total pHH3 cells (*n* = 3 biological replicates) 2-way ANOVA Dunnett’s multiple comparison test ****p < 0.0001. Bars represent mean and standard deviation. **C** Quantification of panel **A** of total pHH3 cells (*n* = 3 biological replicates) 2-way ANOVA Dunnett’s multiple comparison test *p = 0.0117. Bars represent mean and standard deviation. **D** A673 cells were treated with either 1 µM XL413 or 300 nM TAK-931 for 0, 24 or 48 h. Cells were then fixed and stained for DNA content (DAPI). Mitotic events were termed abnormal if they showed signs of anaphase bridge formation, lagging chromosome(s) during anaphase or anaphase/metaphase events with clear signs of more than 2 poles (*n* = 2 biological replicates) 2-way ANOVA **p < 0.01. Bars represent mean and standard deviation. Detailed quantitation and images can be found in Fig. S3**E** A673 cells were treated with 1 µM XL413 or 300 nM TAK-931 for 0, 24, 48 or 72 h. Cells were then fixed and stained for DNA content (DAPI). Micronuclei-containing cells were then quantified (*n* = 3 biological replicates) 2-way ANOVA ****p < 0.0001. Bars represent mean and standard deviation. **F** A673 cells were treated with 1 µM XL413 or 300 nM TAK-931 for 0, 24, 48 or 72 h. Cells were then fixed and stained for yH2AX, and total nuclear fluorescence intensity was calculated using FIJI image software. Representative quantification of 2 biological replicates 2way ANOVA, *p < 0.05, ****p < 0.0001. **G** Cells were treated with 1 µM XL413 (top row) or 300 nM TAK-931 (bottom row) for 0, 24, 48 or 72 h. Total DNA content was stained (PI) and % cells with <2 N DNA content (red) and >4 N DNA content (blue) were quantified using FACS (*n* = 3 biological replicates) Ordinary one-way ANOVA, *p < 0.05, ***p < 0.001, ****p < 0.0001.

The timing of the appearance of cell death markers along with the significant extension of p-CDC2(Y15) upon dTB-release in the DDKi treated cells prompted us to investigate mitotic entry and progression upon DDK inhibition. Unexpectedly, we found that, when treated with TAK-931 or XL413 for 24 h, Ewing cells entered mitosis with under replicated DNA as evidenced by the appearance of pHH3 positive cell populations with sub- 4 N DNA content (Fig. 4A – bottom, red gates & B – right). Importantly, this was not observed in the U2OS cell line (Fig. 4A – top, red gates & B – left). Interestingly, despite the evidence of clear mitotic entry abnormalities in the A673 cells, there did not appear to be any indication of a mitotic arrest as demonstrated by the absence of an increase in pHH3^+^ cells upon TAK-931 or XL413 treatment (Fig. 4C – right). Immunofluorescent imaging of chromosomal DNA revealed that treatment with both XL413 and TAK-931 caused several abnormal mitotic structures in the Ewing sarcoma cells (Fig. 4D and Fig. S3). These included anaphase bridges, lagging chromosomes and anaphase events with >2 poles. TAK-931 treatment elicited the most potent abnormal mitotic phenotype with the most prominent abnormalities being cells with > 2 poles (1.9% in DMSO vs. 17.0% at 24 h and 33.7% at 48 h TAK-31) and anaphase events with clear lagging chromosomes (8.6% in DMSO versus 21.7% at 24 h and 32.5% at 48 h TAK-931). Importantly, these mitotic abnormalities appeared to be more prominent as treatment duration increased (XL413: 37.2% at 24 h vs. 46.0% at 48 h; TAK-931: 46.2% at 24 h versus 61.8% at 48 h).

A likely outcome of abnormal mitotic entry/progression is mitotic catastrophe. Mitotic catastrophe is a mitotic-linked mechanism of induced cell death that occurs as a result of aberrant chromosomal segregation during mitosis [26]. A common symptom of mitotic catastrophe is the appearance of extranuclear DNA, termed micronuclei, in the cytoplasm of affected cells [27, 28]. Micronuclei are thought to be a consequence of the expulsion of damaged or under-replicated or damaged segments of chromosomes that are inherited by a daughter cell [27]. In accordance with this, we observed a significant increase in the proportion of cells that contained micronuclei after treatment with DDKi (>24 h) (Fig. 4E). Another common outcome of mitotic catastrophe is the induction of DNA damage [28]. Again, we observed a marked increase in the level of yH2AX, indicative of DNA double-strand break formation, upon DDK inhibition (>24 h) (Fig. 4F). Interestingly, there was no evidence of aneuploidy in any of the cell lines tested despite the clear evidence of mitotic abnormalities (Fig. 4G). These results indicate that DDK inhibition causes abnormal mitotic progression resulting in DNA damage and the induction of mitotic catastrophe-associated cell death in Ewing sarcoma cells. This is likely the underlying mechanism leading to DDKi-induced apoptosis in these cells.

Together, our findings reveal that Ewing sarcoma cells rely on DDK for proper DNA replication and mitotic division. These results are the first to display sensitivity to DDK inhibition in Ewing sarcoma cells and reveal a novel potential therapeutic entry point for the treatment of the disease.

## Discussion

Ewing sarcoma is an aggressive childhood malignancy with limited treatment options and severe treatment-associated toxicities. A desperate need for the development of novel, less toxic therapeutics is evident. Here, we uncover a novel molecular target, CDC7 kinase (DDK), that has the potential to provide a unique therapeutic entry point for the treatment of the disease. We report that DDK inhibition resulted in a significant reduction in cell viability and induction of apoptosis, specifically in Ewing sarcoma cells. DDKi-sensitivity appeared to be independent of STAG2, TP53, and CDKN2A status as Ewing cell lines with various genetic backgrounds displayed similar sensitivity to TAK-931 treatment. Furthermore, the TP53 WT cell lines TC32 displayed similar cell cycle and apoptotic induction phenotypes as the other Ewing lines tested, further supporting the idea that this sensitivity is independent of TP53 status (Fig. S4). Treatment with DDK inhibitors dramatically reduced the rate of replication and showed a slight but consistent activation of ATR, indicative of RS induction. Also, we found that there was an accumulation of low EdU incorporating cells, specifically in late S-phase, suggesting an inability to properly complete DNA replication. Importantly, we found that, when released from a double thymidine block (dTB) (synchronization at the beginning of S-phase), Ewing sarcoma cells experienced a significantly prolonged S-phase upon DDK inhibition. There was also a pronounced increase in phospho-CDC2 (Y15), suggesting the activation of the G2 checkpoint and mitotic entry delay. Apoptotic markers became evident following prolonged release (>24 h) from a dTB in the presence of DDKi, and the induction of cell death corresponded to mitotic exit and G1 emergence, suggesting improper mitotic progression. In accordance with this, DDK inhibition caused premature mitotic entry and wide-scale mitotic abnormalities in Ewing cells. This was accompanied by the emergence of cells with micronuclei, indicative of mitotic catastrophe. Together, these findings suggest that DDK is required for the faithful and timely completion of DNA replication in Ewing cells and its inhibition may present a viable therapeutic strategy for the treatment of the disease.

The DDKi-induced reduction in replication rates has several potential sources [13–15, 23]. While previous reports have implicated a role for DDK in the full activation of the ATR-CHK1 axis upon replication fork stalling [13, 15], DDK’s main role during S-phase is the activation of replication origins. Therefore, the underlying cause of the reduction in replication rates is likely due to the loss of this function. Ewing tumors are known to harbor large levels of transcription-associated replication stress (reference [9]) that places a heavier burden on the RS response to maintain replication rates. DDK plays a direct role in the suppression of replication stress through its action on ATR-CHK1 function but also plays a somewhat passive role through its involvement in the activation of replication origins [17, 19, 29]. In the event of a stalled replication fork, the replication of the surrounding DNA relies on either the faithful restart of the stalled fork [30] or compensation of replication completion by an adjacent, un-stalled replication fork [18, 19]. The use of adjacent unfired origins, termed dormant origins, allows for the completion of replication of the DNA adjacent to a stalled fork without the need for fork restart [18, 19]. DDK plays a large role in this process through its involvement in replication origin activation [31, 32]. Therefore, it is likely that the inhibition of DDK disallows for replication progression due to a natural accumulation of stalled forks without the ability to utilize dormant origins for their resolution [32, 33]. This means, then, that DDK inhibition will only affect cells that harbor endogenous sources of replication fork stalling. In Ewing cells, this source is likely to be, at least in part, due to the formation of RNAPII-dependent transcription-associated R-loops [9].

Prolonged DDK inhibition resulted in a prolongation of S-phase and a significant delay in mitotic entry followed by the induction of apoptosis. The delay in the mitotic entry was evidenced by an extension of the phosphorylation of CDC2 at tyrosine 15 upon DDK inhibition. This phosphorylation mark is indicative of a WEE1-mediated inhibition of CDC2 function and mitotic entry inhibition [34]. This suggests that, upon DDK inhibition, WEE1 activity prevents further cell cycle progression, limiting DDKi-induced mitotic aberrations in Ewing sarcoma cells. Therefore, it would be interesting to study the combined effects of DDKi + WEE1i on mitotic entry and progression in Ewing sarcoma. Importantly, the induction of apoptosis appeared to be positively correlated with the expression of the late-M/G1 protein CDT1 and inversely correlated with the expression of the mitotic marker phospho-histone H3 (serine 10) (pHH3) suggesting a disruption to mitotic progression and subsequent cell death induction. So, it is likely that the addition of a WEE1 inhibitor will augment the cytotoxic effects of DDKi in Ewing cells by inhibiting mitotic entry delays.

Despite the apparent attempt to delay mitotic entry, we found that DDK inhibition was accompanied by abnormal mitotic entry/progression in Ewing sarcoma cells. Specifically, a significant proportion of mitotic cells appeared to harbor less than 4 N DNA content indicative of premature mitotic entry. In the A673 cells, we observed signs of premature mitotic entry and no signs of a mitotic arrest. Furthermore, we observed signs of aberrant mitotic progression upon DDK inhibition, such as anaphase/telophase cells that had lagging chromosomes, anaphase bridges, and >2 poled divisions. Interestingly, however, we did not observe signs of aneuploidy in any Ewing sarcoma cell line tested. This was surprising as previous studies found that DDKi-sensitive cells accumulate >4 N DNA content upon prolonged treatment with DDK inhibitors [21].

Together, these results strongly suggest that DDK activity is crucial for the maintenance of Ewing cell DNA replication, mitotic entry/progression, and cell viability and its inhibition may be a viable therapeutic strategy for the treatment of the disease.

## Supplementary information

Original Western Blot Images

Supplemental figure legends

Figure S1

Figure S2

Figure S3

Figure S4

## Data Availability

All data in this project is freely available and available as part of this manuscript. Original western blot images and flow cytometry data are included as supplementary material. For any additional requests, please contact the corresponding author.

## References

[1] Ross KA, Smyth NA, Murawski CD, Kennedy JG (2013). The Biology of Ewing Sarcoma. ISRN Oncol.

[2] Grünewald TGP, Cidre-Aranaz F, Surdez D, Tomazou EM, de Álava E, Kovar H (2018). Ewing sarcoma. Nat Rev Dis Prim.

[3] Fisher C (2014). The diversity of soft tissue tumours with EWSR1 gene rearrangements: a review. Histopathology.

[4] Ozaki T (2015). Diagnosis and treatment of Ewing sarcoma of the bone: a review article. J Orthop Sci.

[5] Nieto-Soler M, Morgado-Palacin I, Lafarga V, Lecona E, Murga M, Callen E (2016). Efficacy of ATR inhibitors as single agents in Ewing sarcoma. Oncotarget.

[6] Koppenhafer SL, Goss KL, Terry WW, Gordon DJ (2020). Inhibition of the ATR-CHK1 Pathway in Ewing Sarcoma Cells Causes DNA Damage and Apoptosis via the CDK2-Mediated Degradation of RRM2. Mol Cancer Res: MCR.

[7] Koppenhafer SL, Goss KL, Terry WW, Gordon DJ (2018). mTORC1/2 and Protein Translation Regulate Levels of CHK1 and the Sensitivity to CHK1 Inhibitors in Ewing Sarcoma Cells. Mol. Cancer Therapeutics.

[8] Goss KL, Koppenhafer SL, Harmoney KM, Terry WW, Gordon DJ (2017). Inhibition of CHK1 sensitizes Ewing sarcoma cells to the ribonucleotide reductase inhibitor gemcitabine. Oncotarget.

[9] Gorthi A, Romero JC, Loranc E, Cao L, Lawrence LA, Goodale E (2018). EWS-FLI1 increases transcription to cause R-loops and block BRCA1 repair in Ewing sarcoma. Nature.

[10] Gan W, Guan Z, Liu J, Gui T, Shen K, Manley JL (2011). R-loop-mediated genomic instability is caused by impairment of replication fork progression. Genes Dev.

[11] Moiseeva TN, Yin Y, Calderon MJ, Qian C, Schamus-Haynes S, Sugitani N (2019). An ATR and CHK1 kinase signaling mechanism that limits origin firing during unperturbed DNA replication. Proc Natl Acad Sci USA.

[12] Moiseeva T, Hood B, Schamus S, O’Connor MJ, Conrads TP, Bakkenist CJ (2017). ATR kinase inhibition induces unscheduled origin firing through a Cdc7-dependent association between GINS and And-1. Nat Commun.

[13] Yang, CC, H Kato, M Shindo, and H Masai. Cdc7 activates replication checkpoint by phosphorylating the Chk1-binding domain of Claspin in human cells. Elife. 2019:8:e50796. 10.7554/eLife.50796 PMC6996922 31889509

[14] Tenca P, Brotherton D, Montagnoli A, Rainoldi S, Albanese C, Santocanale C (2007). Cdc7 is an active kinase in human cancer cells undergoing replication stress. J Biol Chem.

[15] Sasi NK, Coquel F, Lin YL, MacKeigan JP, Pasero P, Weinreich M (2018). DDK Has a Primary Role in Processing Stalled Replication Forks to Initiate Downstream Checkpoint Signaling. Neoplasia.

[16] Yamada M, Watanabe K, Mistrik M, Vesela E, Protivankova I, Mailand N (2013). ATR-Chk1-APC/CCdh1-dependent stabilization of Cdc7-ASK (Dbf4) kinase is required for DNA lesion bypass under replication stress. Genes Dev.

[17] Labib K (2010). How do Cdc7 and cyclin-dependent kinases trigger the initiation of chromosome replication in eukaryotic cells?. Genes Dev..

[18] Ge XQ, Jackson DA, Blow JJ (2007). Dormant origins licensed by excess Mcm2-7 are required for human cells to survive replicative stress. Genes Dev.

[19] Blow JJ, Ge XQ, Jackson DA (2011). How dormant origins promote complete genome replication. Trends Biochem Sci..

[20] Zhong Y, Nellimoottil T, Peace JM, Knott SR, Villwock SK, Yee JM (2013). The level of origin firing inversely affects the rate of replication fork progression. J Cell Biol.

[21] Iwai K, Nambu T, Dairiki R, Ohori M, Yu J, Burke K (2019). Molecular mechanism and potential target indication of TAK-931, a novel CDC7-selective inhibitor. Sci Adv.

[22] Awasthi P, Foiani M, Kumar A (2015). ATM and ATR signaling at a glance. J Cell Sci.

[23] Rainey, MD, A Quinlan, C Cazzaniga, S Mijic, O Martella, J Krietsch, et al. CDC7 kinase promotes MRE11 fork processing, modulating fork speed and chromosomal breakage. EMBO Rep. 2020;21:e48920. 10.15252/embr.201948920 PMC7403700 32496651

[24] Riabowol K, Draetta G, Brizuela L, Vandre D, Beach D (1989). The cdc2 kinase is a nuclear protein that is essential for mitosis in mammalian cells. Cell.

[25] Ito S, Ishii A, Kakusho N, Taniyama C, Yamazaki S, Fukatsu R (2012). Mechanism of cancer cell death induced by depletion of an essential replication regulator. PLoS One.

[26] Castedo M, Perfettini J-L, Roumier T, Andreau K, Medema R, Kroemer G (2004). Cell death by mitotic catastrophe: a molecular definition. Oncogene.

[27] Luzhna, L, P Kathiria, and O Kovalchuk, Micronuclei in genotoxicity assessment: from genetics to epigenetics and beyond. Frontiers in Genetics, 2013;4:131. 10.3389/fgene.2013.00131 PMC3708156 23874352

[28] Wilhelm T, Olziersky A-M, Harry D, De Sousa F, Vassal H, Eskat A (2019). Mild replication stress causes chromosome mis-segregation via premature centriole disengagement. Nat Commun.

[29] Yamada M, Masai H, Bartek J (2014). Regulation and roles of Cdc7 kinase under replication stress. Cell Cycle.

[30] Zeman MK, Cimprich KA (2014). Causes and Consequences of Replication Stress. Nat Cell Biol.

[31] Ge XQ, Blow JJ (2010). Chk1 inhibits replication factory activation but allows dormant origin firing in existing factories. J Cell Biol.

[32] Alver RC, Chadha GS, Blow JJ (2014). The contribution of dormant origins to genome stability: from cell biology to human genetics. DNA Repair (Amst.).

[33] Zimmerman KM, Jones RM, Petermann E, Jeggo PA (2013). Diminished origin-licensing capacity specifically sensitizes tumor cells to replication stress. Mol Cancer Res.

[34] Ghelli Luserna di Rorà A, Cerchione C, Martinelli G, Simonetti G (2020). A WEE1 family business: regulation of mitosis, cancer progression, and therapeutic target. J Hematol Oncol.

