# Supplementary material for: CDC7 kinase (DDK) inhibition disrupts DNA replication leading to mitotic catastrophe in Ewing sarcoma
Source: Cell Death Discov. 2022 Feb 26;8:85. doi: 10.1038/s41420-022-00877-x (PMC8882187; doi:10.1038/s41420-022-00877-x)

Figure 1B – XL413 cleaved PARP

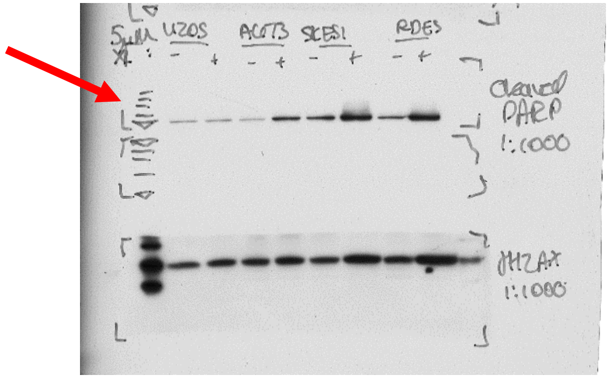

Figure 1B – XL413 GAPDH

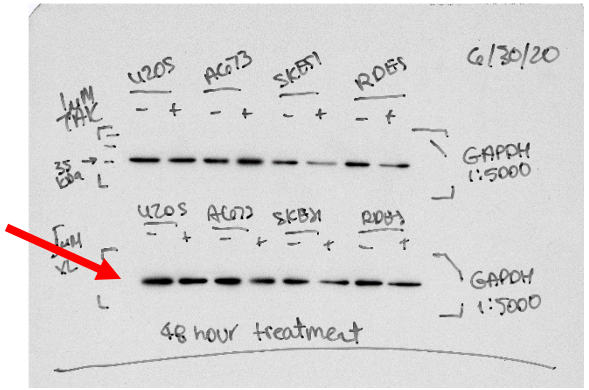

Figure 1B – TAK-931 cleaved PARP and GAPDH

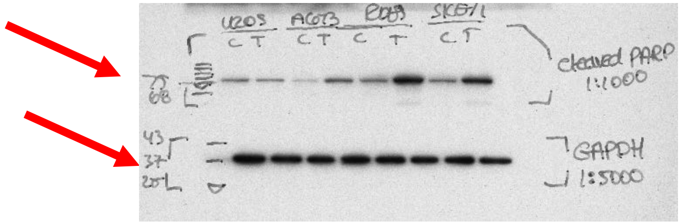

Figure 2A&B – phospho- CHK1 S345

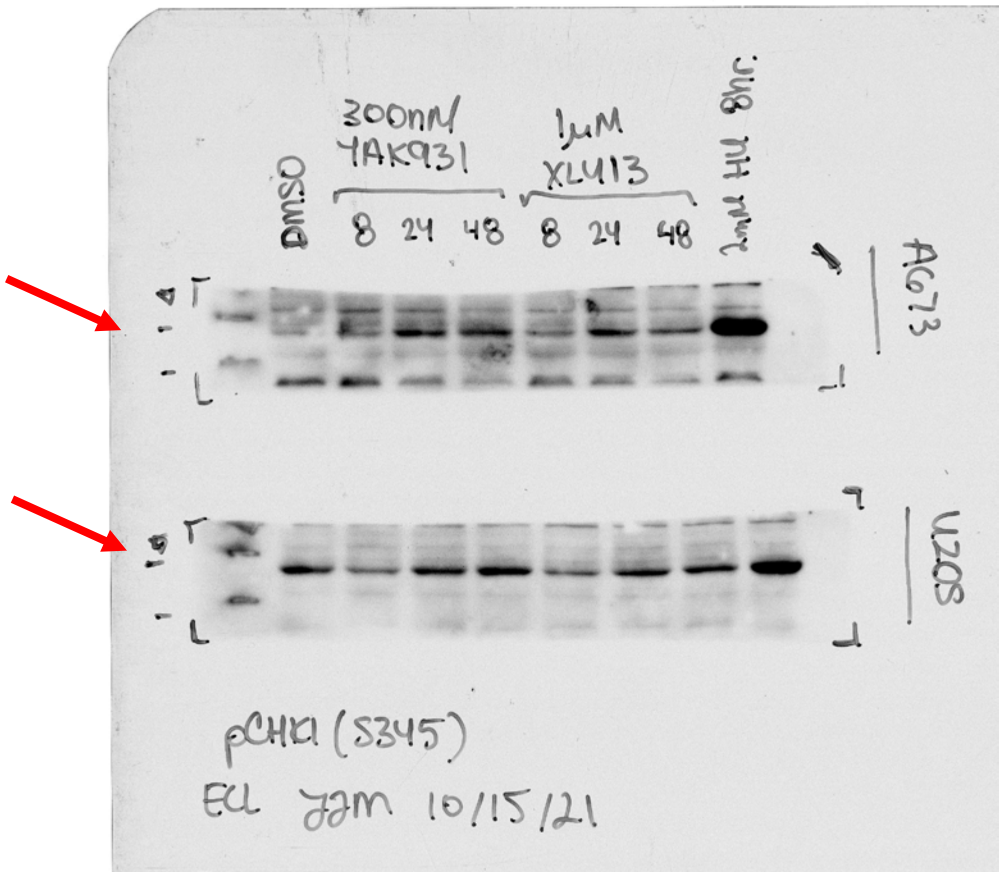

Figure 2B – total CHK1

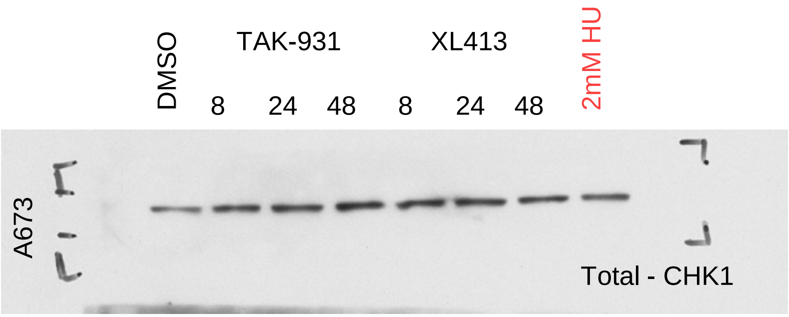

Figure 2B – vinculin

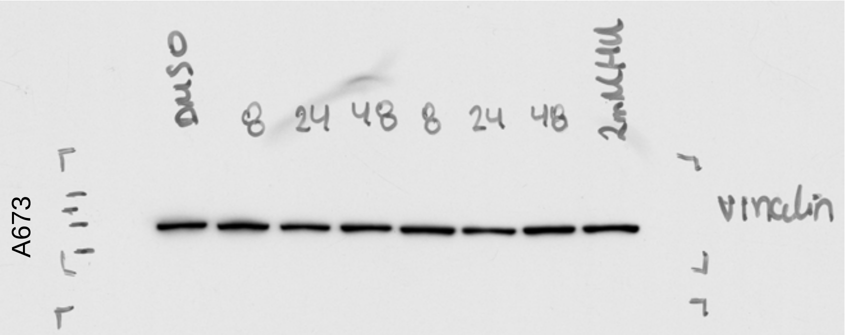

Figure 2B – total cHK1 and vinculin

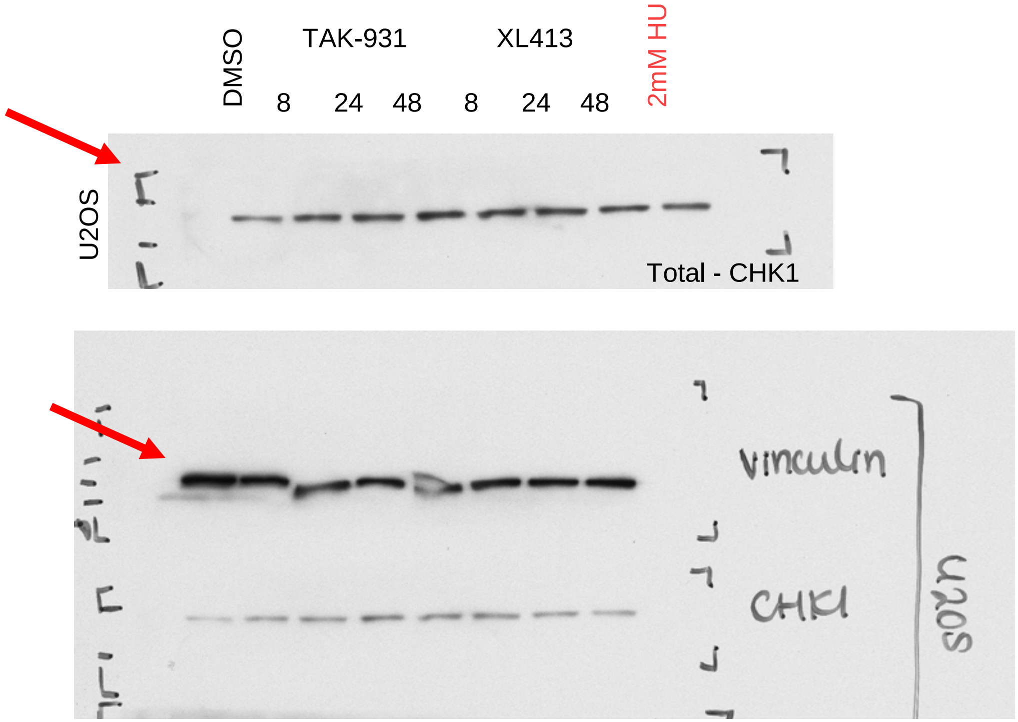

Figure 3B – pHH3 DMSO (left) TAK931 (right)

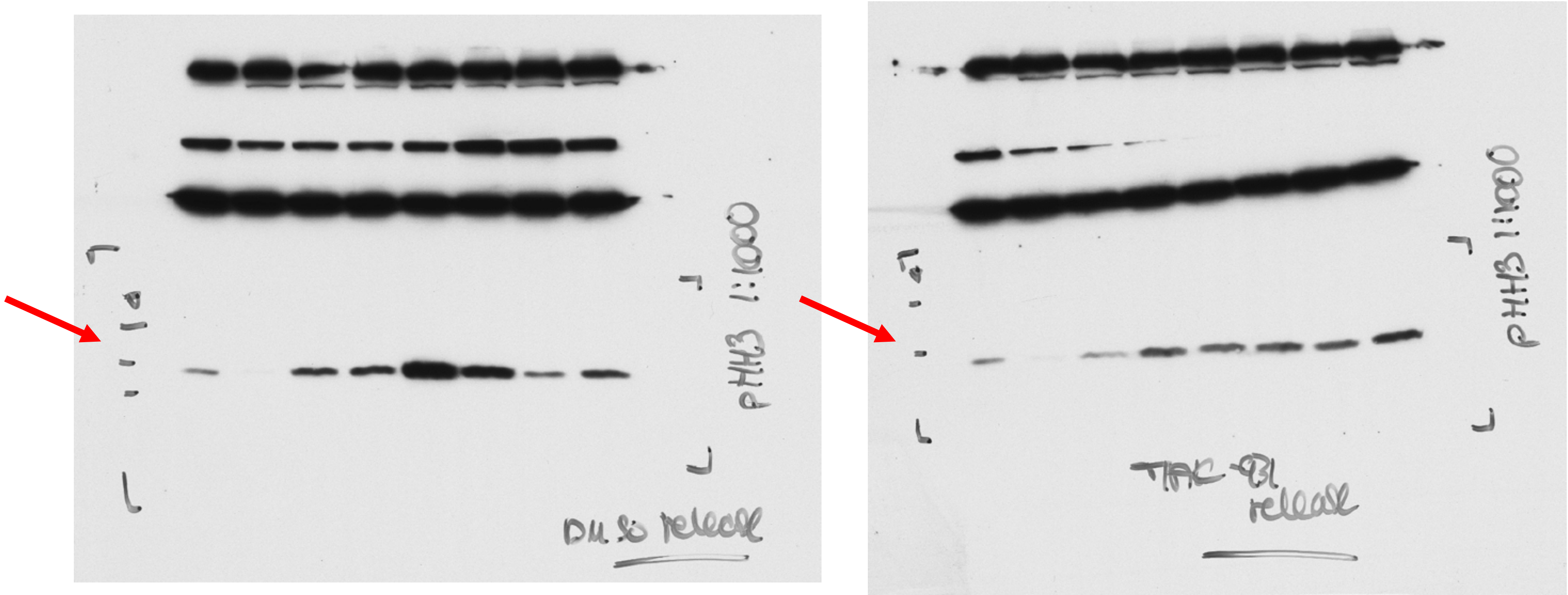

Figure 3B – CDT1 - DMSO (left) TAK931 (right)

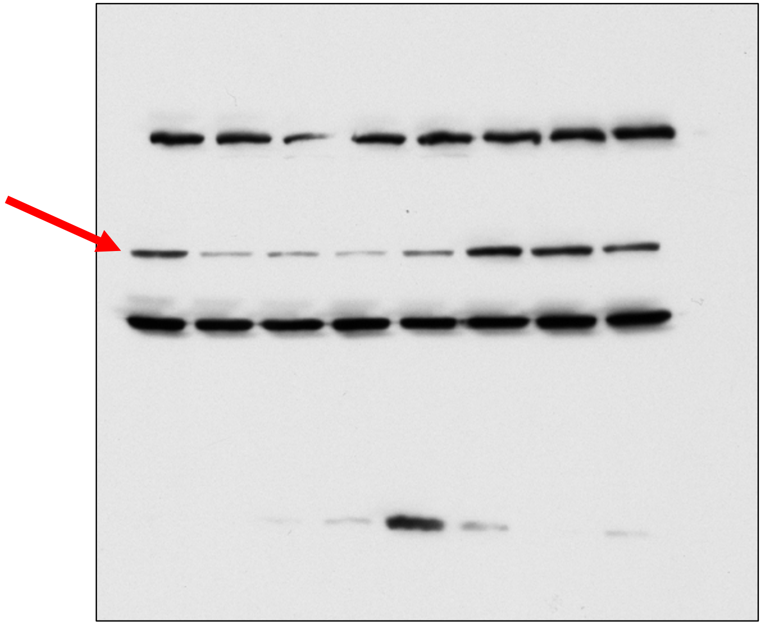

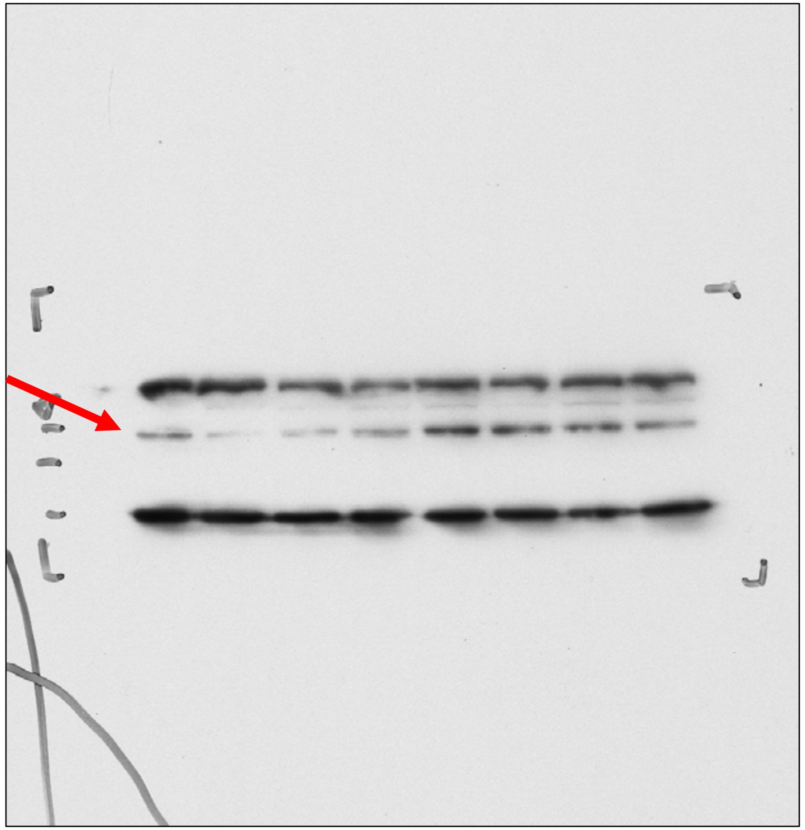

Figure 3B pCDC2, CyclinA and PARP1

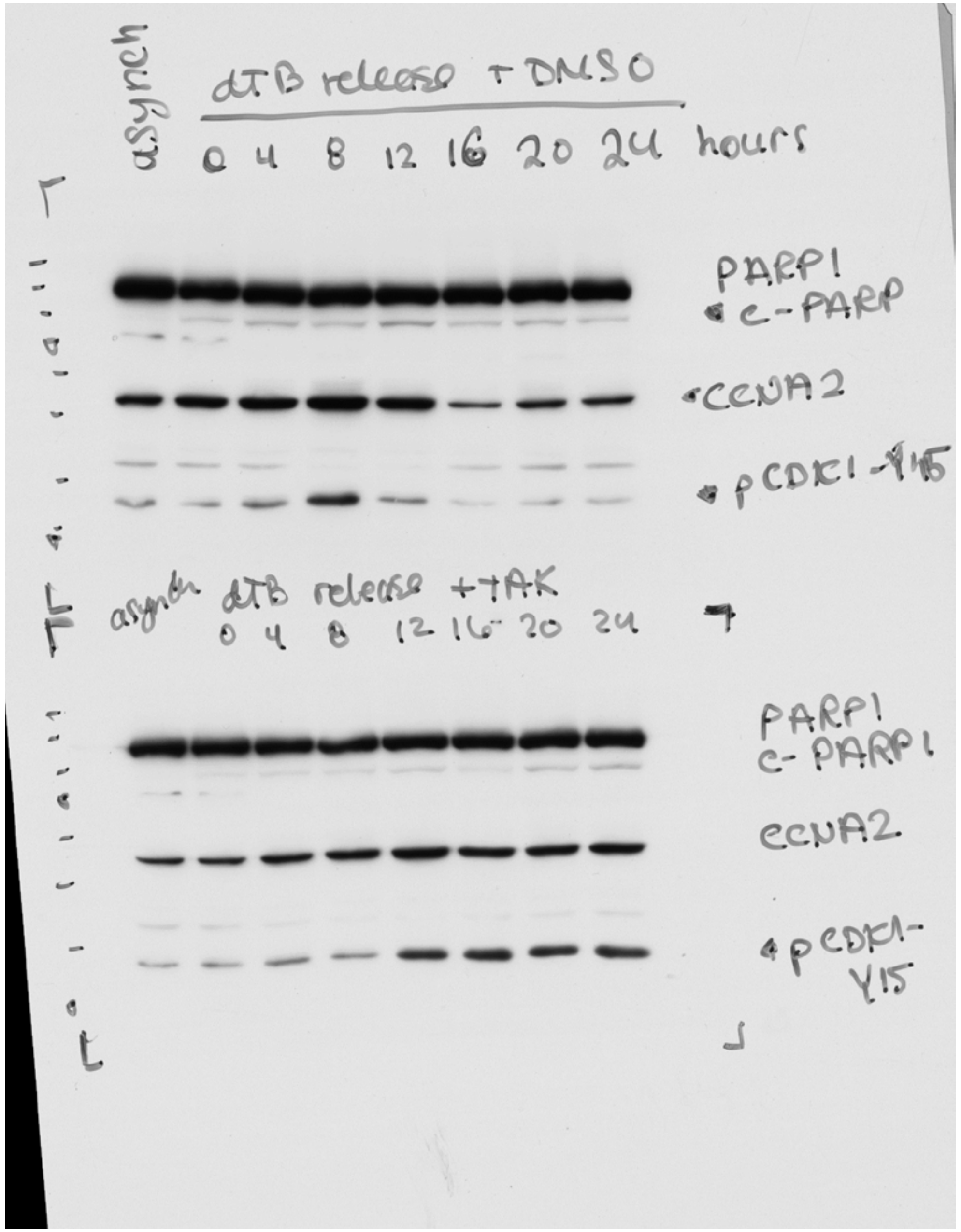

Figure 3B

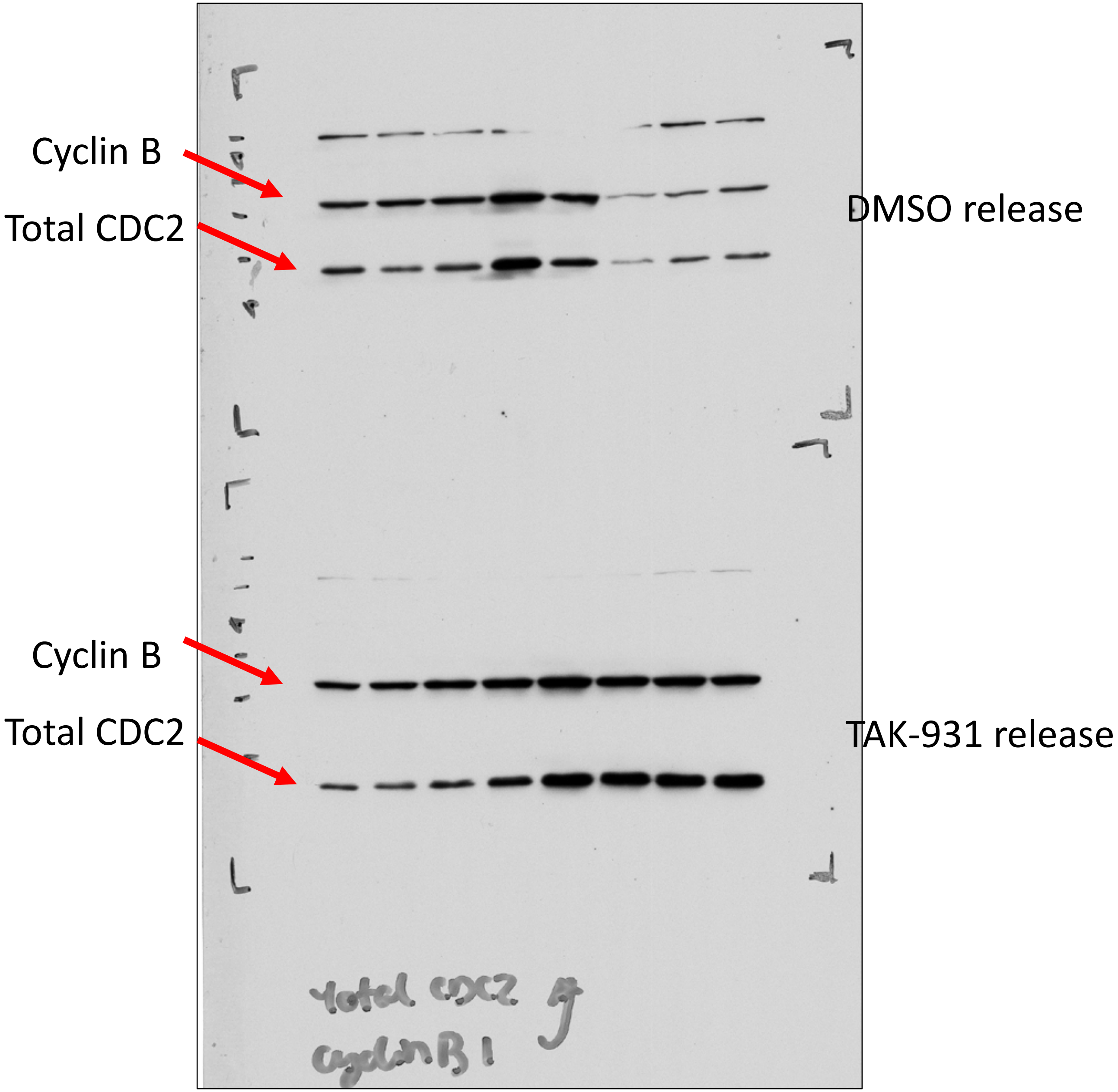
Cyclin B
Total CDC2
DMSO release
Cyclin B
Total CDC2
TAK-931 release

Figure 3B - GAPDH

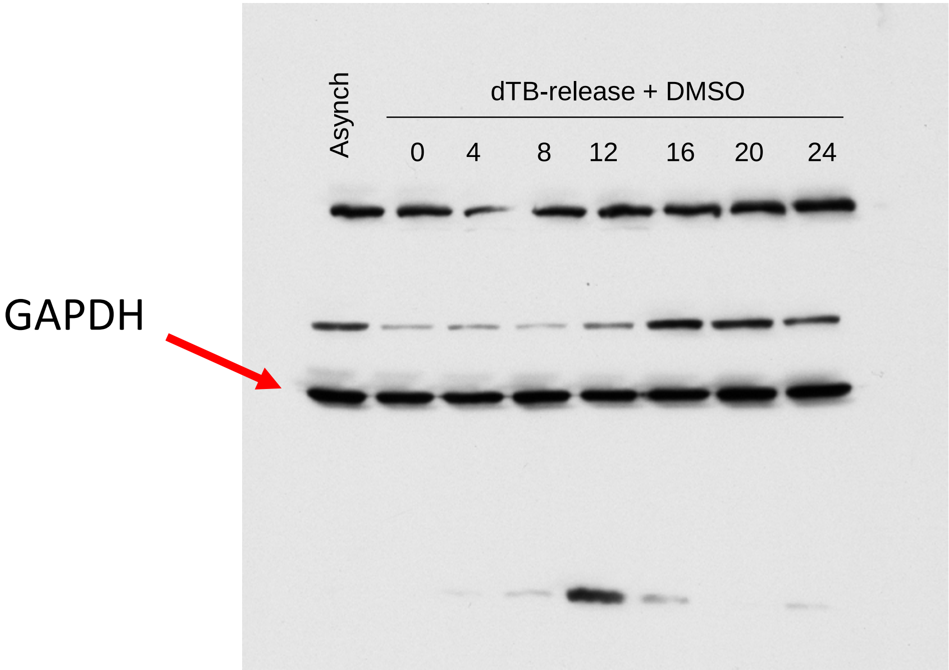

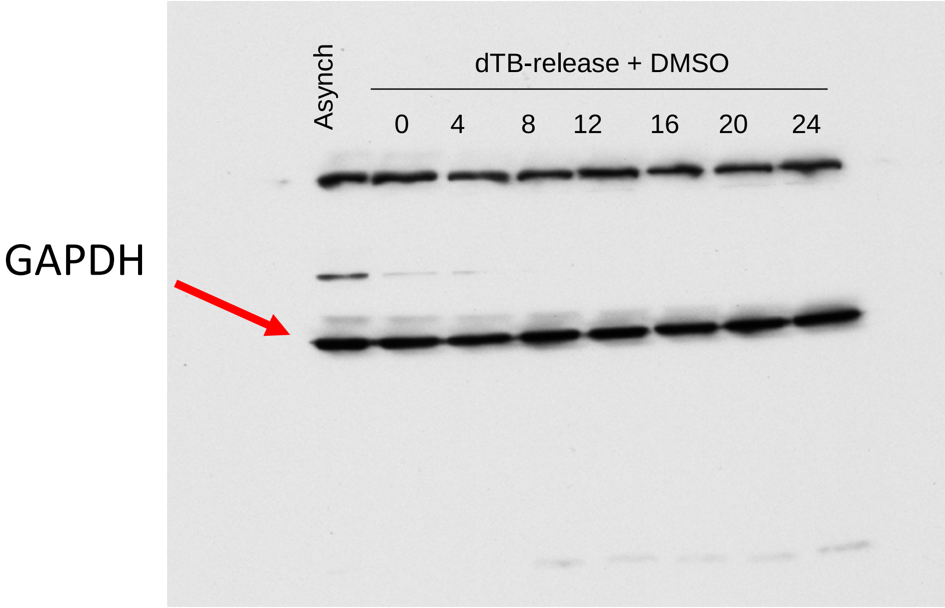

Figure 3C – p-CDC2

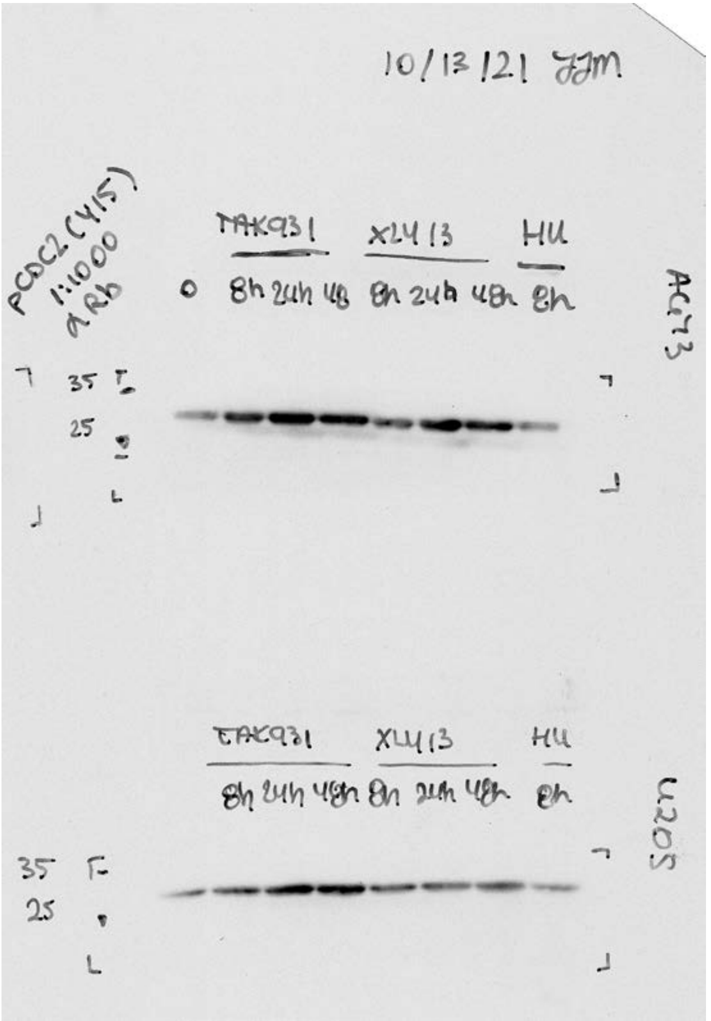

Figure 3C – total CDC2 and vinculin

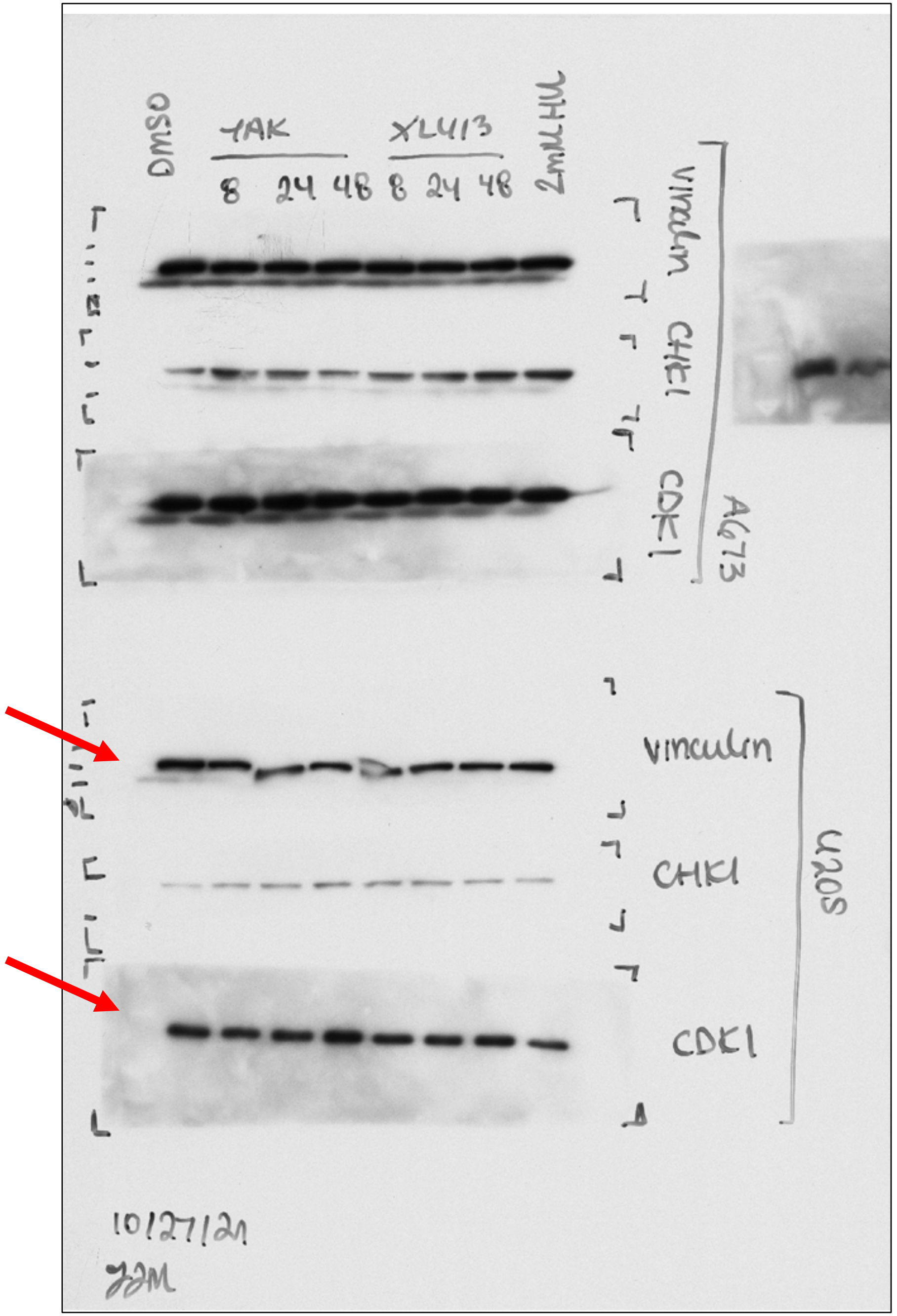

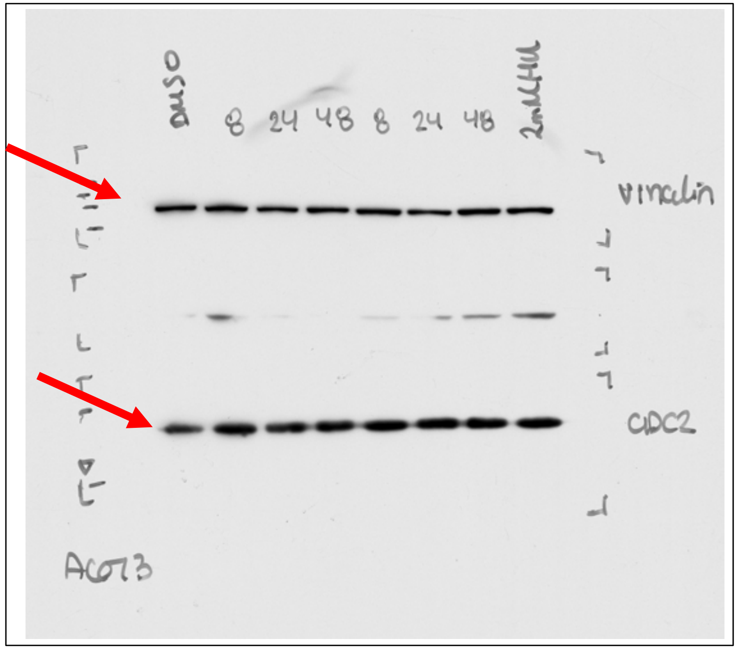

Figure 3D – PARP1 and CDT1

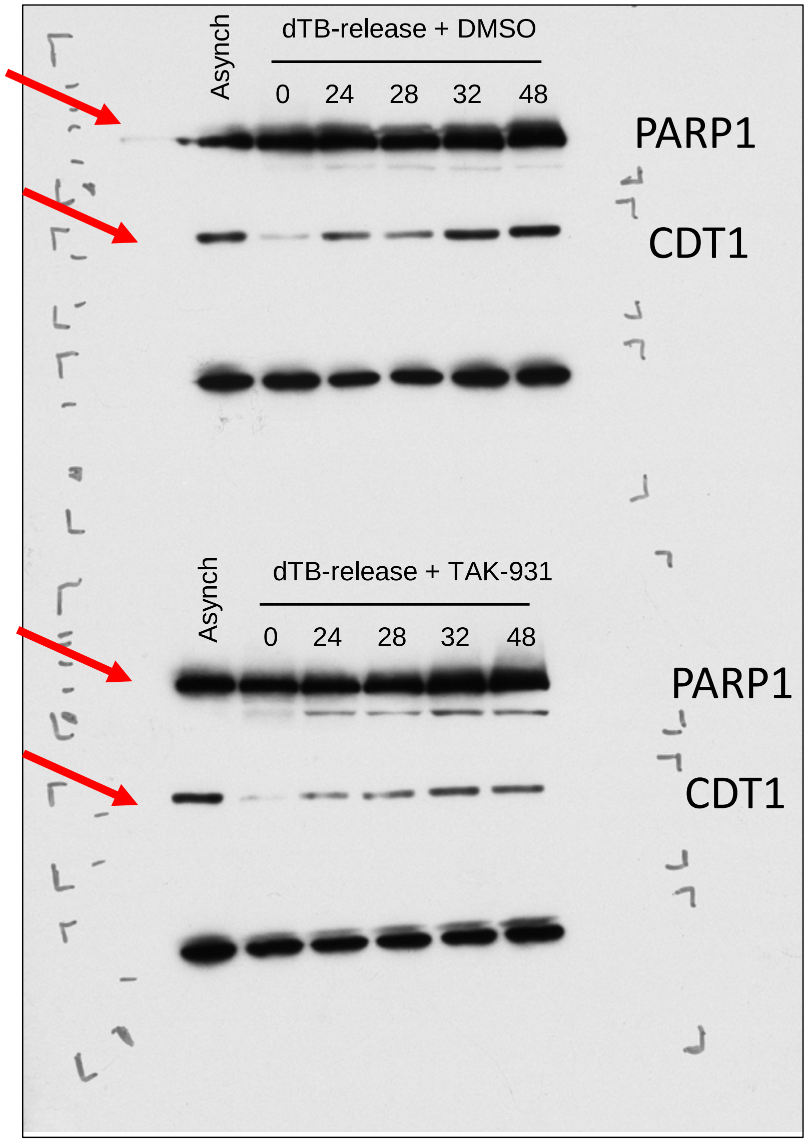

Figure 3D – pHH3

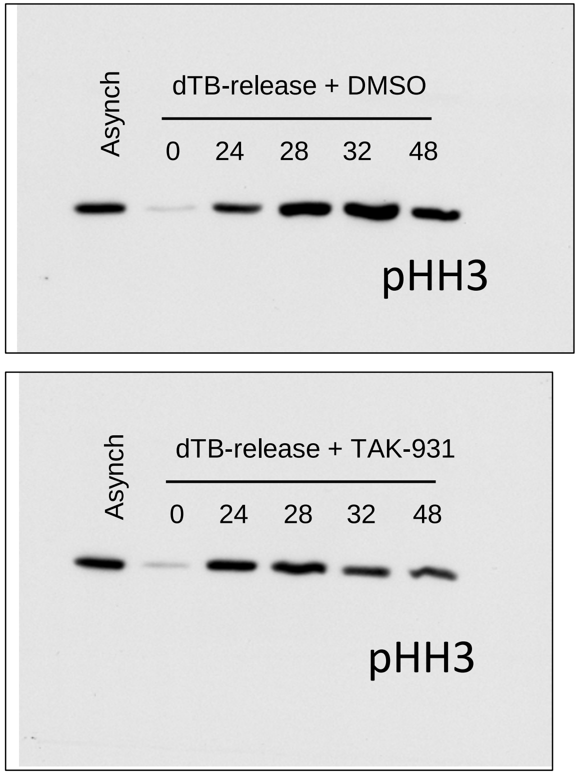

Figure 3D – cleaved caspase 3

C-caspase 3
TAK-931 release

C-caspase 3
DMSO release

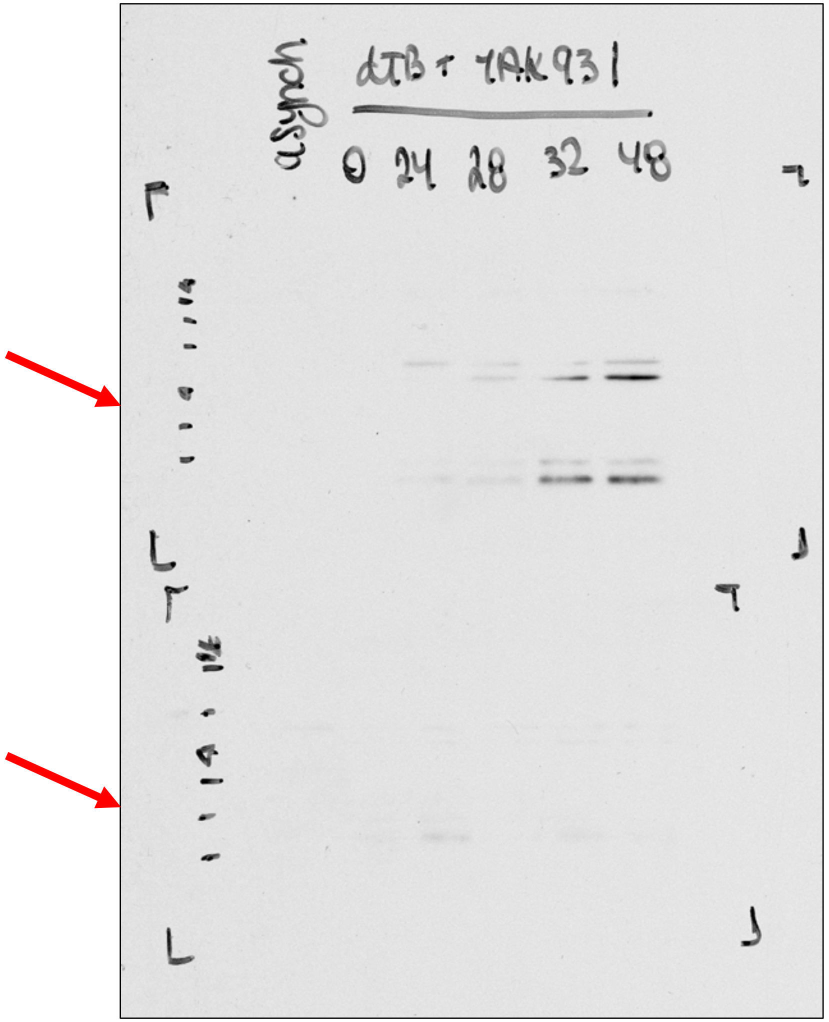

Figure 3D - GAPDH

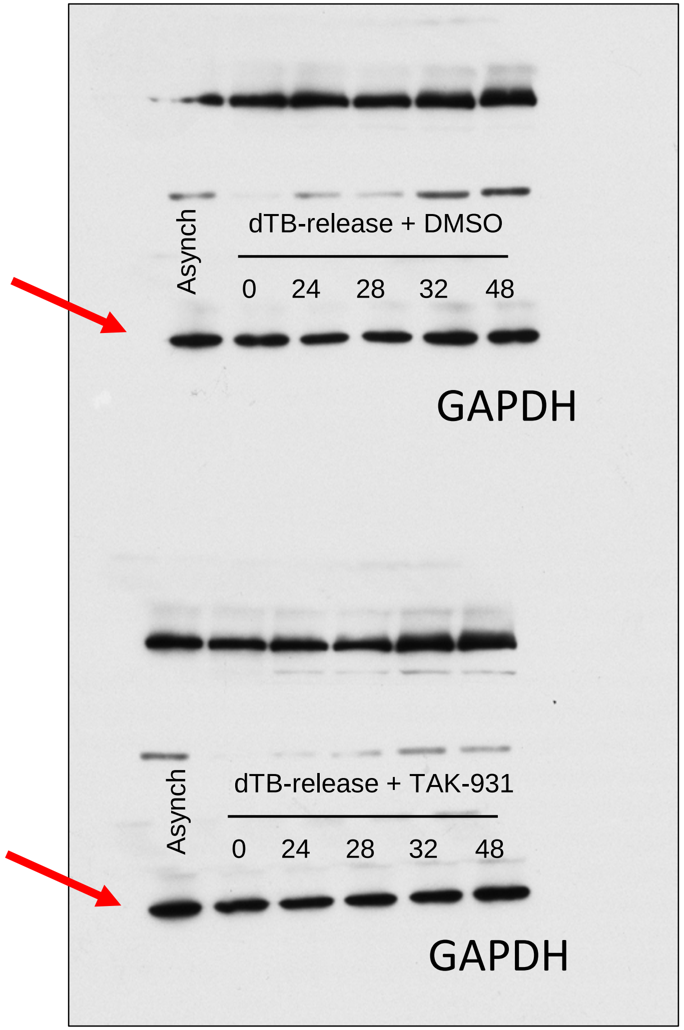

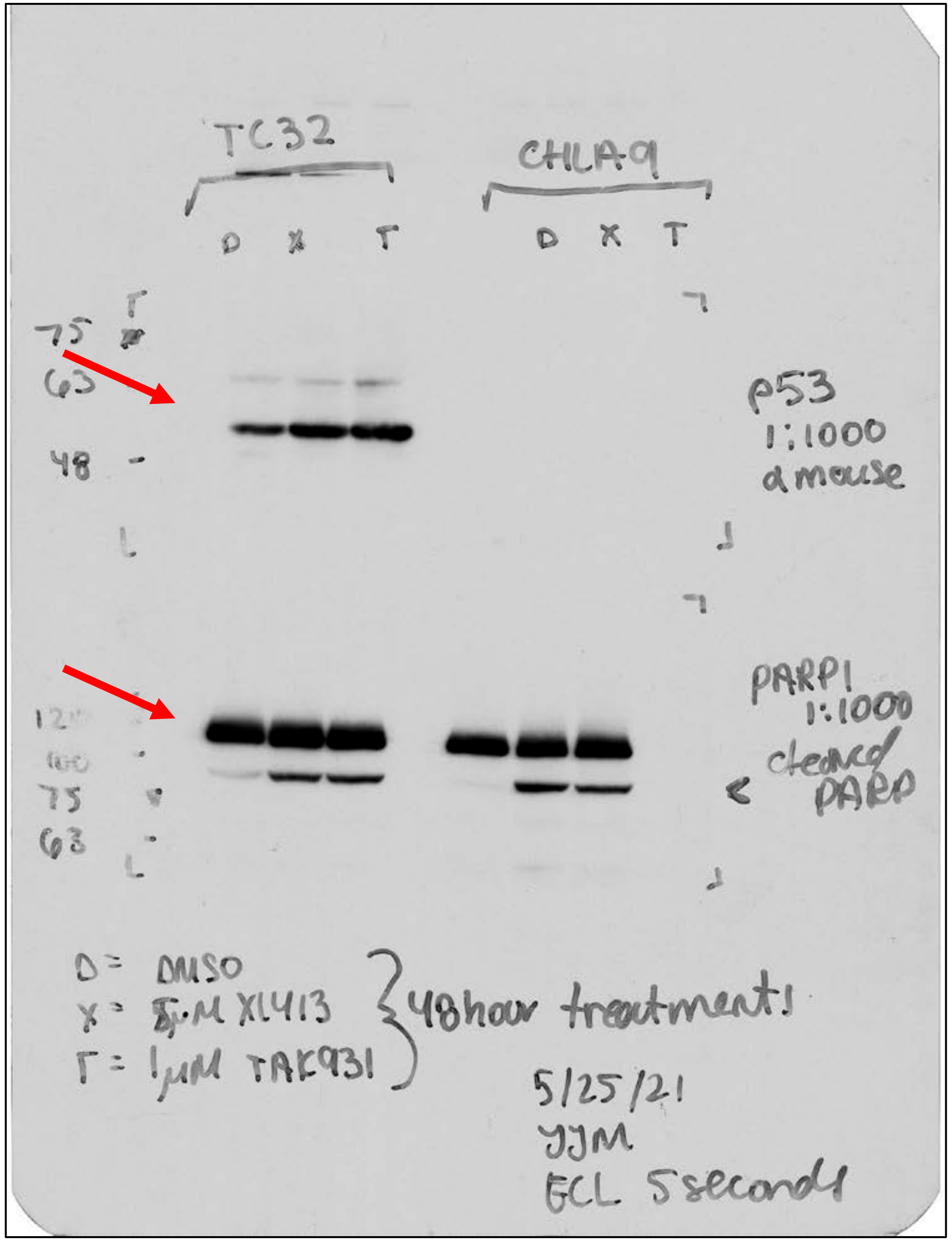

Supplemental figure 4

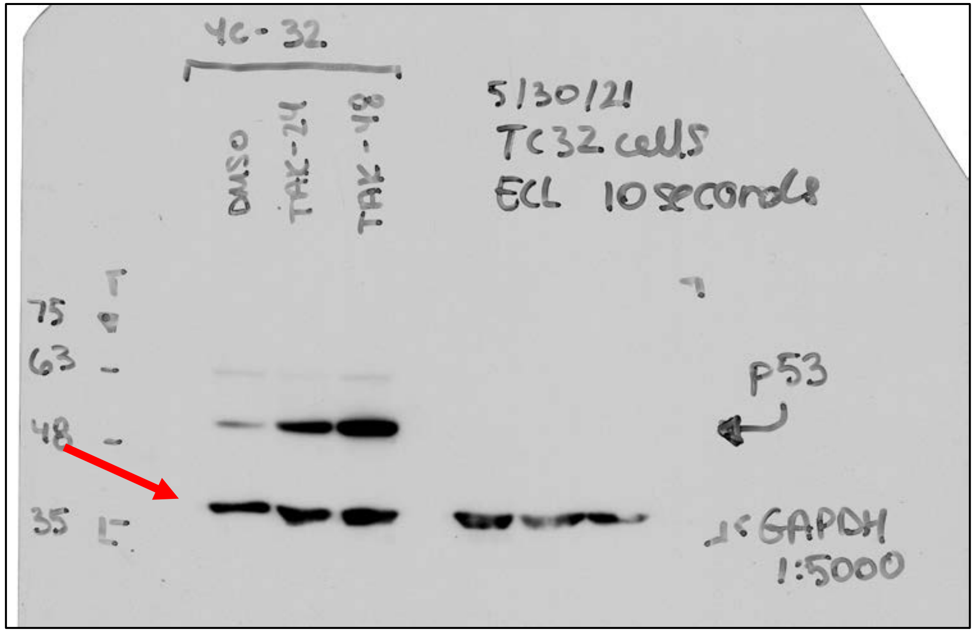

Supplement: Supplementary file 1 — Original Western Blot Images [file 41420_2022_877_MOESM1_ESM.pdf]
