# Supplementary material for: CDC7 kinase (DDK) inhibition disrupts DNA replication leading to mitotic catastrophe in Ewing sarcoma
Source: Cell Death Discov. 2022 Feb 26;8:85. doi: 10.1038/s41420-022-00877-x (PMC8882187; doi:10.1038/s41420-022-00877-x)
Supplement: Supplementary file 2 — Supplemental figure legends [file 41420_2022_877_MOESM2_ESM.docx]

**Supplemental Figure S1. Ewing sarcoma cells are sensitive to the DDK inhibitor TAK-931 intendent of TP53. STAG2 and CDKN2A status.** A) A panel of Ewing sarcoma cell lines (colored lines) and the osteosarcoma cell line U2OS (black line) were treated with a wide range of doses of TAK-931 for 72 hours. Cell viability was assessed using CCK8 reagent and relative viability was calculated based on DMSO treated control cells. B) IC50 values were calculated using GraphPad prism software (non-linear regression [Inhibitor] vs. Response – Variable slope (four parameters)) for TAK-931 based on relative viability curves generated in panel A. Green bars represent all Ewing sarcoma cell lines and black bar is U2OS non-Ewing control. C, D & E) IC50 values were compared between cell lines of different genetic backgrounds (TP53, STAG2 and CDKN2A status). C) Cell lines were arranged from based on TAK-931 sensitivity from most sensitive (top) to least sensitive (bottom). A colored grid was then generated using red squares to represent WT gene status and blue squares to represent altered (mutant/deleted) gene status. No apparent trends were observed. D) Red dots represent IC50 values of cells with WT STAG2 and blue dots represent IC50 values of cells with mutant STAG2. E) Red dots represent IC50 values of cells with WT TP53 and blue dots represent dots IC50 values of cells with mutant TP53.

**Supplemental Figure S2. DDK inhibition causes aberrant cell cycle accumulation in Ewing Sarcoma cells.** Ewing Sarcoma cells and U2OS cells were treated with 0.1% DMSO (72 hours), 300nM TAK-931 or 1µM XL413 for 24, 48 and 72 hours, DNA was stained with propidium iodide and DNA content was analyzed using flow cytometry. Cell cycle distribution was measured using FCS Express v7. A) Representative histograms of three independent experiments B) Quantification of cell cycle distribution; G1 = grey bars, S = white bars, G2/M = blue bars, sub-G1 = red bars (n=3 biological replicates).

**Supplemental Figure S3. DDK inhibition causes abnormal mitotic progression in Ewing sarcoma cells (extended).** A673 cells were treated with either 1µM XL413 or 300nM TAK-931 for 0, 24 or 48 hours. Cells were then fixed and stained for DNA content (DAPI). Mitotic events were termed abnormal if they showed signs of anaphase bridge formation, lagging chromosome(s) during anaphase or anaphase/metaphase events with clear signs of more than 2 poles (n = 2 biological replicates).

**Supplemental Figure S4. TP53-WT TC32 Ewing sarcoma cells are sensitive to DDKi.** A) TC32 cells were treated with specified concentrations of DDKi - XL413 (left- grey bars) and TAK-931 (right – black bars) for 72 hours and cell viability was assessed using CCK8. Relative viability was calculated based on DMSO treated controls (n = 3 technical triplicates; Ordinary one-way ANOVA compared to DMSO treated control ****p<0.0001). B) TC32 cells were treated with either 0.1% DMSO, 5µM XL413 or 1µM TAK-931 for 48 hours. Protein lysate was collected, and a western blot was run for specific proteins. GAPDH was used as a loading control (n = 1). C) TC32 cells were treated with 300nM TAK-931 for specified times (DMSO = 0.1% DMSO for 72 hours). DNA content histograms were generated using flow cytometry and cell cycle distributions were quantified in panel D.
