# Supplementary figures and images for: CDC7 kinase (DDK) inhibition disrupts DNA replication leading to mitotic catastrophe in Ewing sarcoma
Source: Cell Death Discov. 2022 Feb 26;8:85. doi: 10.1038/s41420-022-00877-x (PMC8882187; doi:10.1038/s41420-022-00877-x)

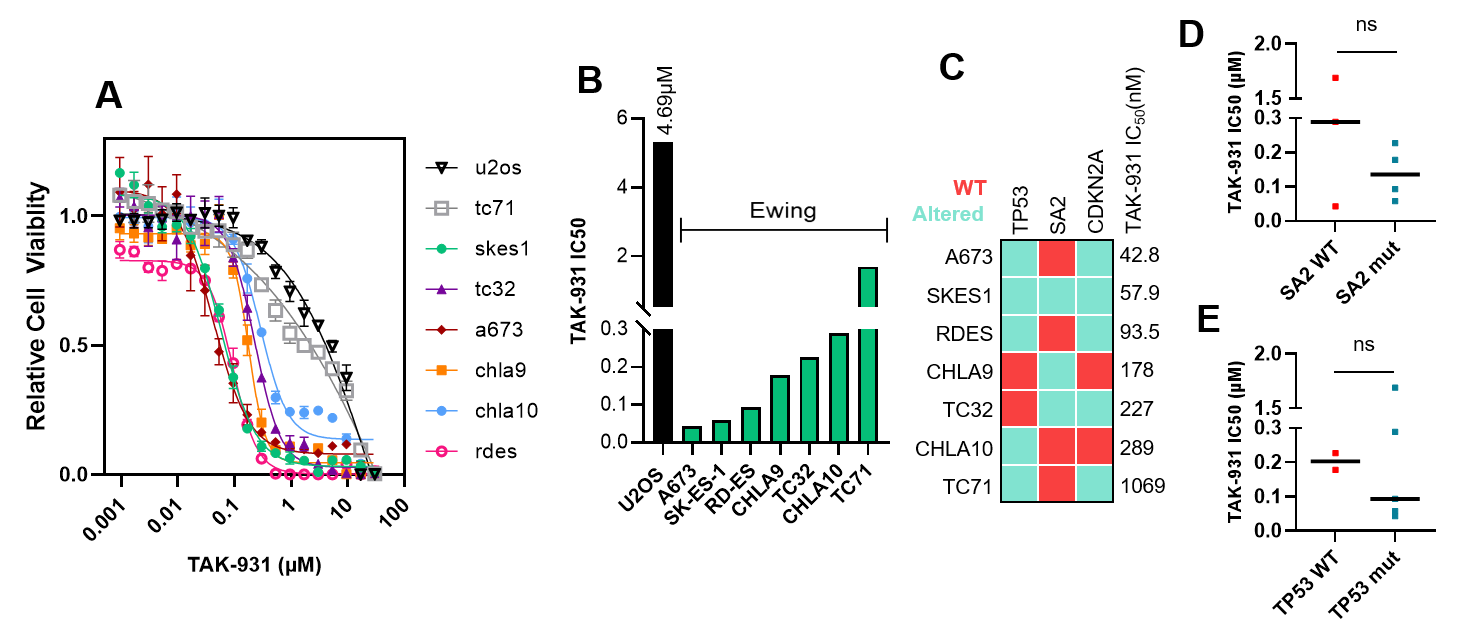

Supplement: Supplementary file 3 — Figure S1 [file 41420_2022_877_MOESM3_ESM.png]

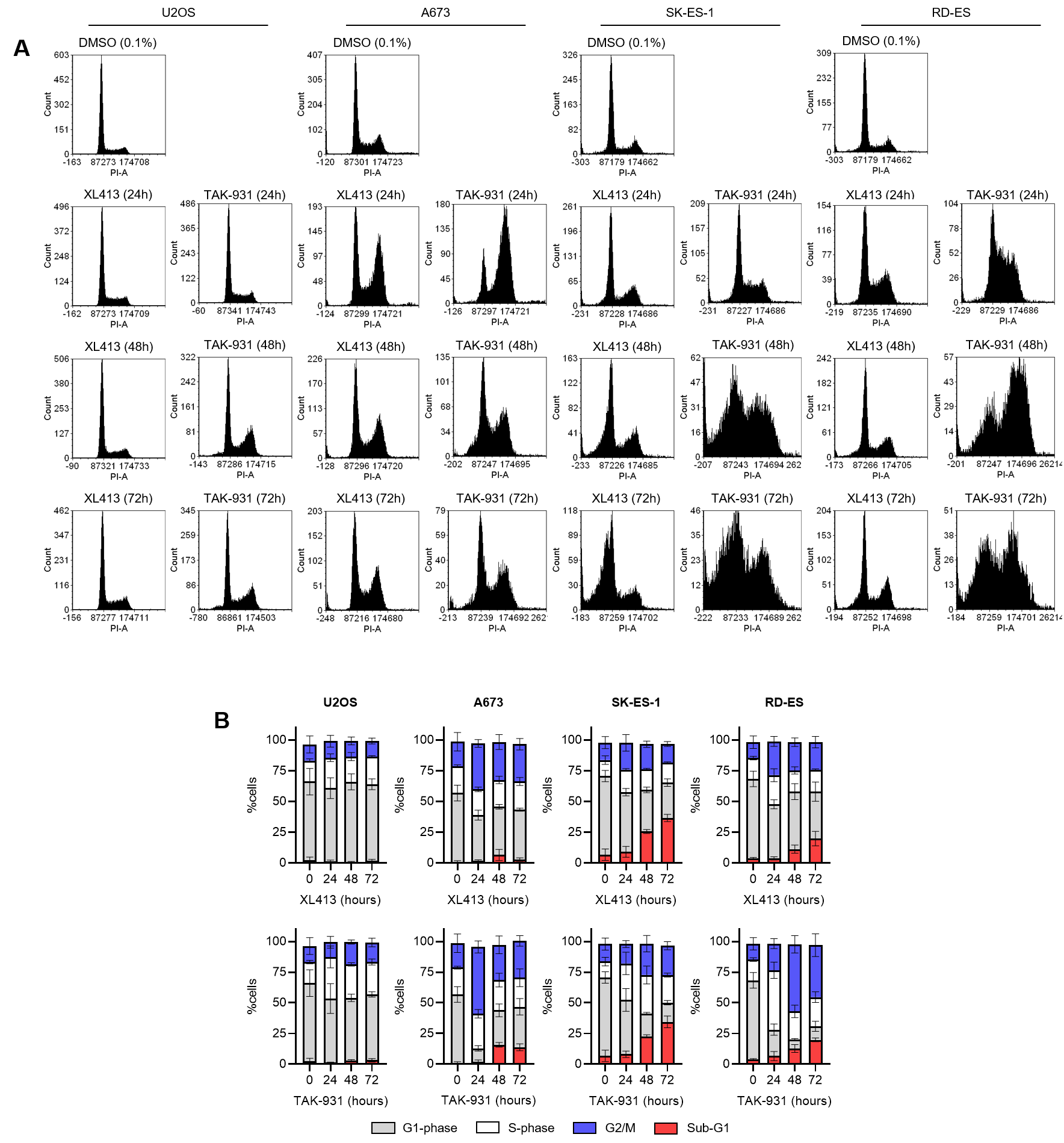

Supplement: Supplementary file 4 — Figure S2 [file 41420_2022_877_MOESM4_ESM.png]

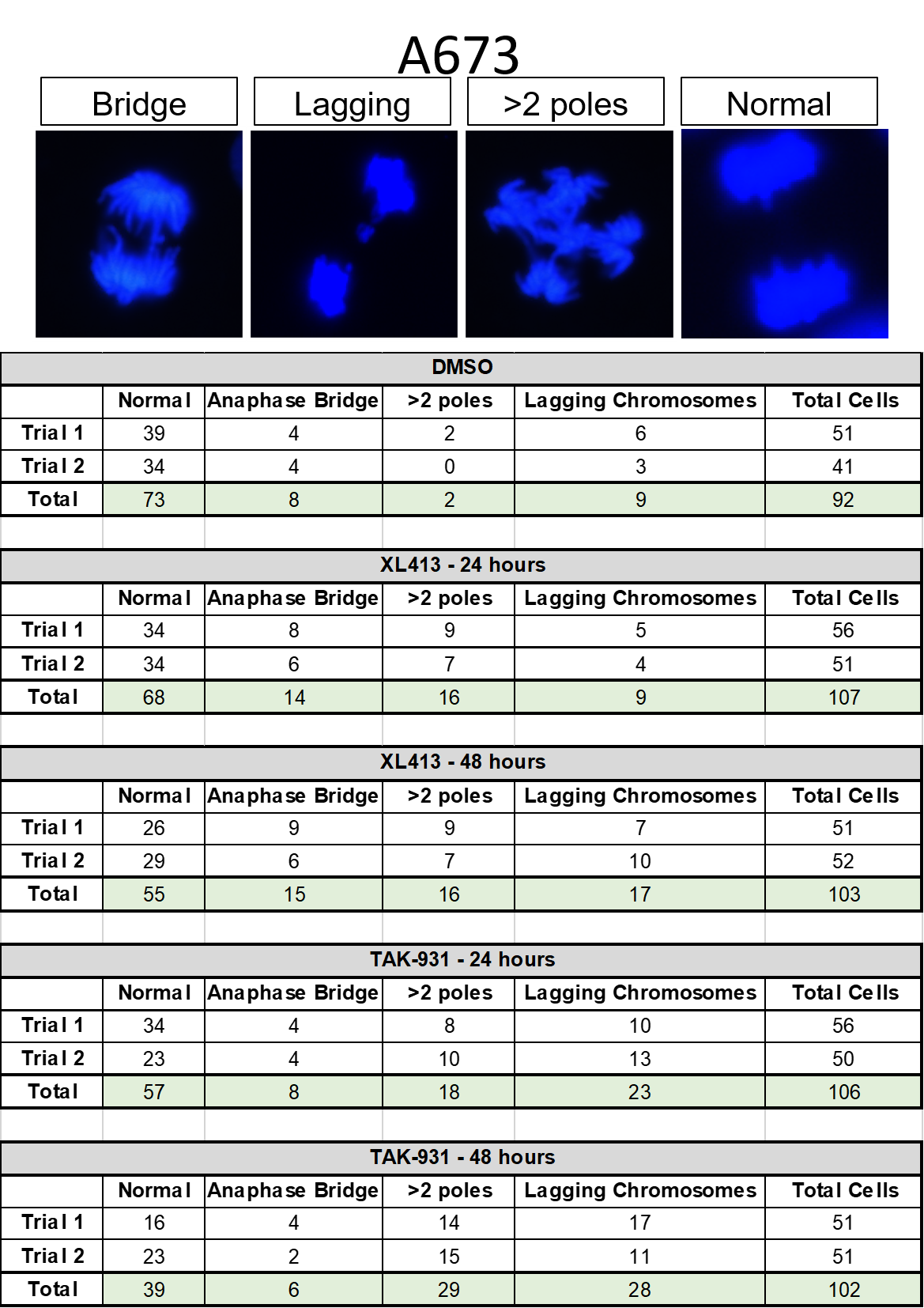

Supplement: Supplementary file 5 — Figure S3 [file 41420_2022_877_MOESM5_ESM.png]

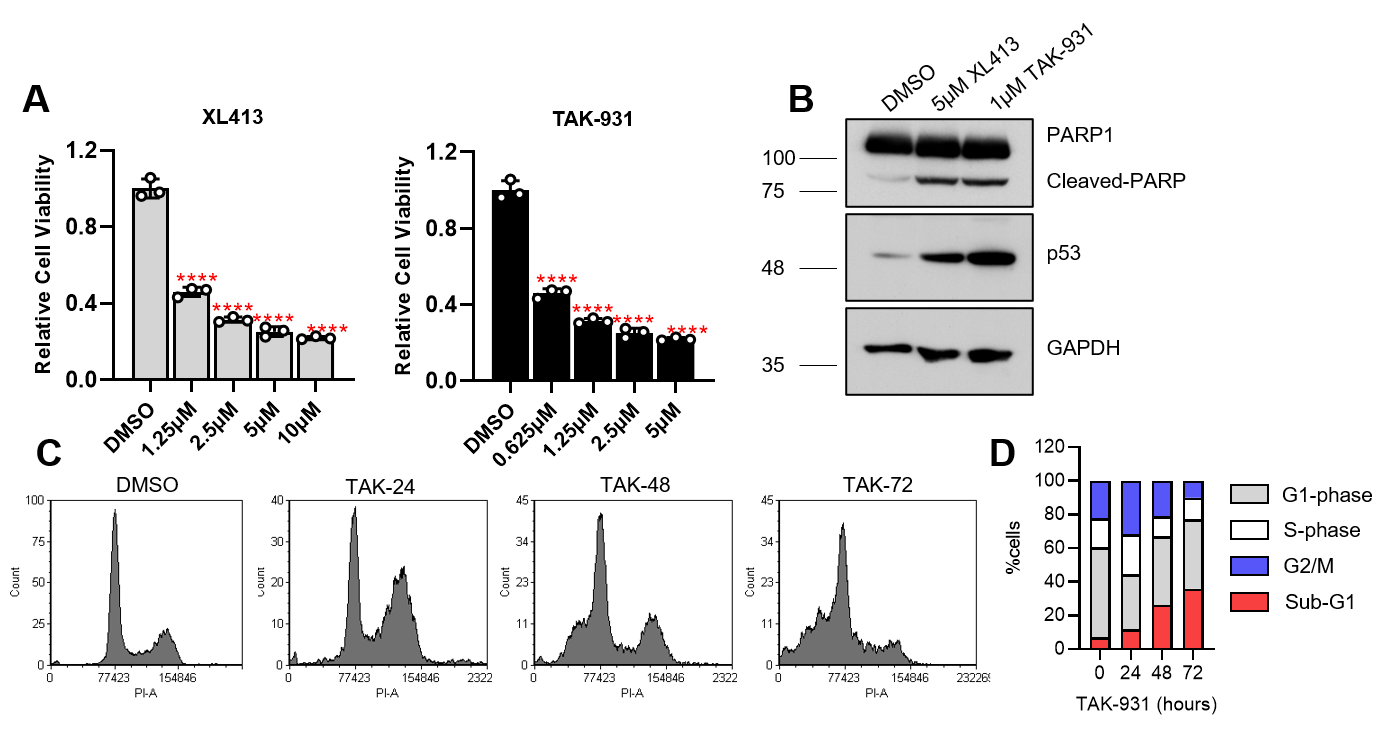

Supplement: Supplementary file 6 — Figure S4 [file 41420_2022_877_MOESM6_ESM.png]
